# Starfysh integrates spatial transcriptomic and histologic data to reveal heterogeneous tumor–immune hubs

**DOI:** 10.1038/s41587-024-02173-8

**Published:** 2024-03-21

**Authors:** Siyu He, Yinuo Jin, Achille Nazaret, Lingting Shi, Xueer Chen, Sham Rampersaud, Bahawar S. Dhillon, Izabella Valdez, Lauren E. Friend, Joy Linyue Fan, Cameron Y. Park, Rachel L. Mintz, Yeh-Hsing Lao, David Carrera, Kaylee W. Fang, Kaleem Mehdi, Madeline Rohde, José L. McFaline-Figueroa, David Blei, Kam W. Leong, Alexander Y. Rudensky, George Plitas, Elham Azizi

**Affiliations:** 1 Department of Biomedical Engineering, Columbia University, New York, NY USA; 2 Irving Institute for Cancer Dynamics, Columbia University, New York, NY USA; 3 Department of Computer Science, Columbia University, New York, NY USA; 4 Pharmaceutical Sciences and Pharmacogenomics Graduate Program, University of California, San Francisco, San Francisco, CA USA; 5 Immunology Program, Sloan Kettering Institute, Memorial Sloan Kettering Cancer Center, New York, NY USA; 6 The Graduate School of Biomedical Sciences at the Icahn School of Medicine at Mount Sinai, New York, NY USA; 7 Department of Biomedical Engineering, Washington University in St. Louis, St. Louis, MO USA; 8 Department of Pharmaceutical Sciences, University at Buffalo, the State University of New York, Buffalo, NY USA; 9 Department of Computer Science, Fordham University, New York, NY USA; 10 Briarcliff High School, New York, NY USA; 11 Herbert Irving Comprehensive Cancer Center, Columbia University, New York, NY USA; 12 Department of Statistics, Columbia University, New York, NY USA; 13 Department of Systems Biology, Columbia University Irving Medical Center, New York, NY USA; 14 Howard Hughes Medical Institute, Memorial Sloan Kettering Cancer Center, New York, NY USA; 15 Ludwig Center, Memorial Sloan Kettering Cancer Center, New York, NY USA; 16 Department of Surgery, Breast Service, Memorial Sloan Kettering Cancer Center, New York, NY USA; 17 Data Science Institute, Columbia University, New York, NY USA

**Keywords:** Machine learning, Computational models

## Abstract

Spatially resolved gene expression profiling provides insight into tissue organization and cell–cell crosstalk; however, sequencing-based spatial transcriptomics (ST) lacks single-cell resolution. Current ST analysis methods require single-cell RNA sequencing data as a reference for rigorous interpretation of cell states, mostly do not use associated histology images and are not capable of inferring shared neighborhoods across multiple tissues. Here we present Starfysh, a computational toolbox using a deep generative model that incorporates archetypal analysis and any known cell type markers to characterize known or new tissue-specific cell states without a single-cell reference. Starfysh improves the characterization of spatial dynamics in complex tissues using histology images and enables the comparison of niches as spatial hubs across tissues. Integrative analysis of primary estrogen receptor (ER)-positive breast cancer, triple-negative breast cancer (TNBC) and metaplastic breast cancer (MBC) tissues led to the identification of spatial hubs with patient- and disease-specific cell type compositions and revealed metabolic reprogramming shaping immunosuppressive hubs in aggressive MBC.

## Abstract (web summary)

Reference-free integrative analysis of spatial transcriptomics data is achieved with archetype analysis.

## Main

In multicellular organisms, the function of diverse cell types is strongly influenced by their surroundings. Uncovering the spatial organization and communication between cell types in tissues provides insight into their development, response to stimuli, adaptations to their microenvironment or transformation into malignant or diseased states^1^. By sampling the entire transcriptome, ST has enabled unbiased gene expression mapping in a spatially resolved manner, providing an opportunity to study the spatial arrangement of cells and microenvironments^2^. These technologies have been employed in diverse fields, including organ development, disease modeling and immunology^3–5^. However, sequencing-based methods (Visium, DBiT-seq^6^, Slide-seq^7^ and so on) are limited in cellular resolution due to technical limitations, including artifacts from lateral RNA diffusion^2^. Hence, measurements from capture locations (spots) involve mixtures of multiple cells, leading to analytical challenges in dissecting the cellular disposition, particularly in complex cancerous tissues.

Accurate characterization of cell types and refined states is critical for comparing their spatial organization and communication across tissues. This is essential, for example, when studying changes in cellular wiring during development or disease progression. In tumor tissues, the mixing of signals from patient-specific tumor cells and immune cells hinders the comparison of anti-tumor immune mechanisms between patients or disease subtypes. Most existing computational methods for analyzing ST data (Cell2location^8^, DestVI^9^, Tangram^10^, Stereoscope^11^, RCTD^12^, BayesPrism^13^ and so on) require paired and annotated single-cell data as references to overcome this challenge and are not capable of integrating tissue samples. The references, whether from the same tissue or public databases, could introduce biases without accounting for sample or batch variation and variable cell density across spots. Indeed, using a single-cell atlas reference has been shown to increase deconvolution error compared to reference-free approaches^14^.

Importantly, access to paired single-cell data may not be cost-effective or practical, especially in cases like clinical core biopsies. This limitation further motivates the development of reference-free methods capable of integrating prior knowledge of cell type markers and data from multiple tissues to improve statistical power. Reference-free methods including STdeconvolve^14^, Smoother^15^ and CARD^16^ deconvolve spots into latent factors. However, some factors cannot be explicitly mapped to refined cell states in complex tissues. Additionally, these methods are not scalable and do not allow the integration of multiple ST datasets. Batch correction methods designed for single-cell RNA sequencing (scRNA-seq) are also not feasible in integrating ST samples dominated by sample-specific cell types such as tumor cells. While some methods use histology images to align spots between replicate tissues^8^ or predict high-resolution gene expression from histology, they fail to leverage spatial dependencies and paired histology to improve cell state deconvolution.

To address this need, we developed a comprehensive toolbox for multimodal analysis and integration of ST datasets dubbed ST analysis using reference-free deep generative modeling with archetypes and shared histology (Starfysh). With joint modeling of transcriptomic measurements and histology images, Starfysh infers the proportion of fine-grained and context-dependent cell states while obtaining cell type-specific gene expression profiles for downstream analysis. Integration of gene expression and histology accounts for tissue architecture, cell density, structured technical noise and spatial dependencies between measurements, which improve the characterization of cell states and their arrangement. By integrating multiple tissues, Starfysh identifies shared or sample-specific niches and underlying cell–cell crosstalk.

The innovation of our machine learning approach is in incorporating archetypal analysis and known cell type markers as priors within a deep generative model that maps transcriptomic features and histology from multiple tissues to a joint latent space. Archetypes, which capture spots with the most different expression profiles, construct or refine cell type markers, in contrast to conventional clustering of spots, which obtain markers corresponding to aggregated cell types^17^. Archetypes empower Starfysh to characterize new or context-specific cell states and present a hierarchy among them.

Starfysh shows successful, robust deconvolution without requiring single-cell references on simulated data and accurately recapitulated cell state proportions in breast tumor datasets^18^. Additionally, we profiled tumor samples from ER^+^ patients, patients with TNBC and patients with MBC to demonstrate Starfysh’s utility for spatial mapping of intertumoral and intratumoral heterogeneity and studying the role of microenvironmental niches in determining localized immune response. Starfysh’s archetypal analysis characterized patient-specific tumor cell states and their spatial arrangement within the primary tumor, revealing how the underlying biology of tumor states and environmental signals alters the immune response. We further identified metabolic reprogramming and communication enriched in the rare and aggressive MBC subtype by integrating our data with previously published ST datasets. Starfysh thus presents a powerful analytical platform for systematic interrogation and comparative studies of complex tissues in health and disease through the lens of ST and histology.

## Results

### Starfysh performs reference-free deconvolution of cell types

**Fig. 1:**
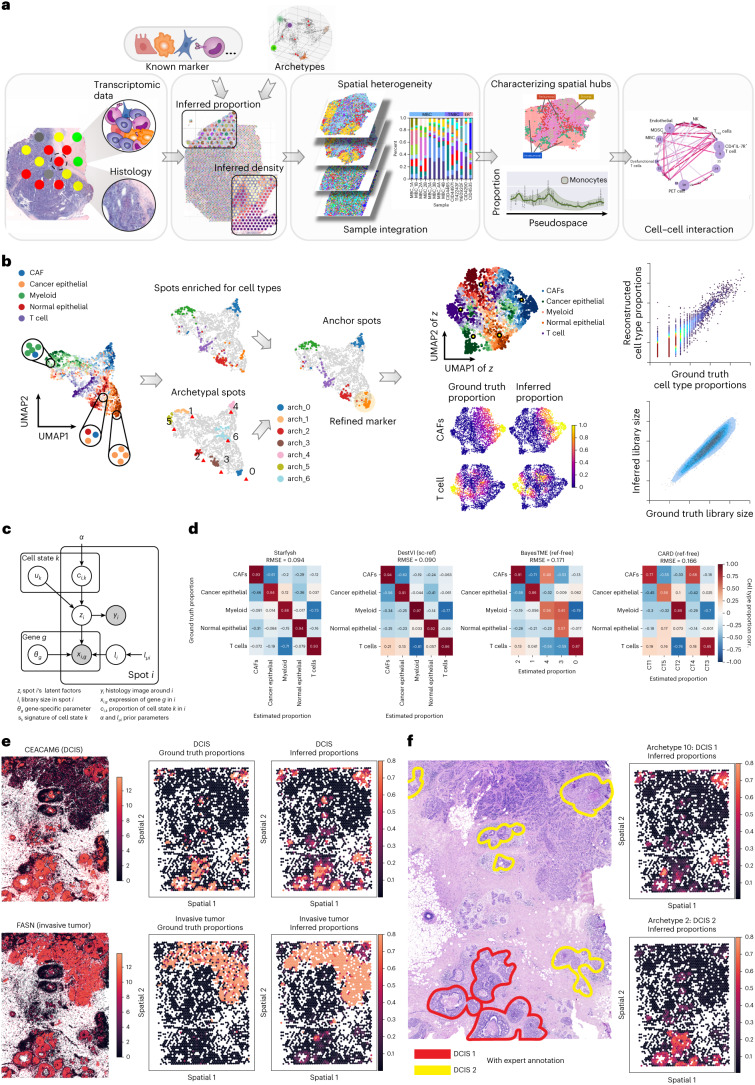
Starfysh overview and performance on simulated data. **a**, Overview of the Starfysh workflow. From left to right: Starfysh input (ST dataset, signature gene lists for cell types or cell states and paired histology image (optional)), deconvolution (Starfysh defines anchor spots representative of cell types or states with the aid of archetypal analysis and infers cell type or state proportions and densities by accounting for ST technical artifacts), sample integration and downstream analysis (upon deconvolution, Starfysh jointly integrates multiple samples and characterizes spatial ‘hubs’ and further infers cell–cell interactions within each hub). NK, natural killer; PET, peripheral T. **b**, Left: UMAP of ST data with 2,500 spots, 29,631 genes and 5 cell types simulated from mixtures of scRNA-seq data of breast tumor tissues, colored by the proportion of most enriched cell type in the ground truth. Starfysh collectively uses signature gene sets and archetypal analysis to identify anchor spots, refine marker gene sets and discover potential new cell states. Right: comparison of ground truth cell type proportions and densities in simulated data and the Starfysh reconstruction (Methods and Supplementary Fig. 2a). **c**, Graphical representation of the deep generative model integrating transcriptomic data and paired histology images to infer a joint latent space. **d**, Benchmarking Starfysh against other methods on the simulated dataset: Pearson correlation of ground truth and estimated proportions per cell type in data. The performance of each method is summarized by computing the average root-mean-squared error (RMSE) across spots against the ground truth (Methods). Additional benchmarking and robustness analysis results are shown in Supplementary Fig. 2c–e. Benchmarking on real breast tumor ST data is shown in Supplementary Fig. 3a–d (Methods). corr., correlation; ref-free, reference free; sc-ref, reference with scRNA-seq data. **e**, Spatial distribution of marker expression from breast tumor Xenium data used for generating spot-level ground truth to compare to inferred proportions from Starfysh applied to matched Visium data (Methods). DCIS and invasive tumor marker and cell types are shown. Other cell types and details are shown in Supplementary Fig. 4a–e. CEACAM6, CEA cell adhesion molecule 6; FASN, fatty acid synthase. **f**, Expert annotations of two distinct subsets of DCIS (red and yellow) are aligned with Starfysh-predicted archetypes (without the use of signatures that distinguish them).

Starfysh is an end-to-end toolbox for multimodal analysis and integration of ST datasets (Fig. 1a). In short, Starfysh features reference-free deconvolution of cell types and fine-grained cell states, enhanced by integrating paired histology images, if available. To facilitate the comparison of tissues, Starfysh identifies common or sample-specific spatial ‘hubs’, defined as niches with a unique composition of cell states. To uncover mechanisms underlying cell communication, Starfysh conducts downstream analyses of these hubs and identifies key spatially variable genes, cell states and colocalization networks.

To circumvent the need for matched or external single-cell references, Starfysh leverages two key concepts to determine spots with the most distinct expression profiles as ‘anchors’ that pull apart and decompose spots in the latent space (Fig. 1b). First, Starfysh incorporates a compendium of known or custom cell state marker gene sets. Assuming that spots with the highest expression of a gene set corresponding to a cell state are likely to have the highest proportion of that cell state, these spots form an initial set of anchors. Second, because cell state markers can be context dependent or not well characterized, Starfysh uses archetypal analysis to adapt the anchors. Archetypes can also discover new cell states and their hierarchical relationships (Methods). This feature is paramount in characterizing context-specific cell states, for example, patient-specific tumor cells, their phenotypic plasticity and dynamic crosstalk within the microenvironment.

Inspired by successful implementations of deep generative models in single-cell omics analysis (scvi-tools^19^, scVI^20^, totalVI^21^, scArches^22^, trVAE^23^, scANVI^24^, MrVI^25^), Starfysh jointly models ST and histology as data observed from a shared low-dimensional latent representation while incorporating anchors as priors. Specifically, we define latent representations of spots as mixtures of cell states guided by anchors (Fig. 1c, Supplementary Fig. 1a and Methods). To test the performance of Starfysh, we simulated ST data from real scRNA-seq data from primary breast tumor tissues^18^ with different levels of cell type granularity (Supplementary Fig. 1b–d and Methods). Starfysh successfully recovered cell type proportions and cell density (Fig. 1b and Supplementary Fig. 2a–e).

Starfysh integrates histology to correct for artifacts in transcriptomic measurements by considering spatial dependencies between spots and incorporating tissue structure, which improves cell density estimation and neighborhood characterization in complex tissues. The integration of two data modalities is accomplished using the product of experts (PoE^26^), which calculates the joint posterior distribution for gene expression and images (Fig. 1c and Methods). We simulated ST data with spatial dependencies using a Gaussian process model^8^ and simulated images by training a ResNet18 (ref. ^27^) encoder followed by a variational autoencoder on paired ST expression and histology images (Supplementary Fig. 1c and Methods). Simulated ST data harbored cell clumps and histology patterns resembling real tissues (Supplementary Fig. 2a). The PoE integrates latent factors from transcriptomic and histology data and shows significantly improved performance in predicting the proportion of cell types and reconstructing high-density regions (Supplementary Fig. 2b). We benchmarked Starfysh against existing tools and found the deconvolution performance of Starfysh to be comparable to state-of-the-art methods that require a single-cell reference including DestVI^9^, Cell2location^8^, Tangram^10^ and BayesPrism^13^ (Fig. 1d). Additionally, compared to reference-free methods such as CARD^16^, BayesTME^28^ and STdeconvolve^14^, Starfysh shows a significant improvement in deconvolving both major and finer cell types (Supplementary Fig. 2d,e; Mann–Whitney *U*-test, *P* < 1 × 10^−5^). Applied to published ST data from a TNBC breast tumor sample (patient CID44971)^18^, Starfysh also shows substantial improvement in disentangling fine-grained cell states (Mann–Whitney *U*-test, *P* = 1.70 × 10^−11^) and scalability compared to other methods (Supplementary Fig. 3a–g and Methods).

We further validated the assumptions and performance of Starfysh with archetypal analysis using a recent breast tumor ST dataset and matched single-cell RNA in situ Xenium data^29^. The multicellular-resolution ST spots were mapped to single cells annotated by Xenium profiling through image registration (Methods). Starfysh outperforms other reference-free methods: given the same input signature gene sets from this public dataset, Starfysh obtained an improved deconvolution for major cell types matching Xenium profiles (Supplementary Fig. 4a–f). We also used these data to confirm that archetypes detect ‘purest spots’, that is, dominant in one cell type (Supplementary Fig. 5a,b). In fact, archetypal analysis guided Starfysh to delineate refined cell states of ductal carcinoma in situ (DCIS) without prior knowledge of markers distinguishing them: archetypes 10 and 2 correspond to expert-annotated subtypes DCIS 1 (low grade) and DCIS 2 (high grade) respectively, whereas competing reference-free methods failed to recover them (Fig. 1e,f and Supplementary Fig. 5b,c).

As an illustration of generalizability to other tissue types, Starfysh successfully decomposed cell types and delineated the spatial microenvironment in the mouse brain and human lymph nodes (Supplementary Fig. 6a–f), recapitulating the findings of Cell2location, which uses a single-cell reference^8^. In addition to dissecting single tissues, Starfysh was capable of integrating ST data from a diverse cohort of prostate cancer and tracking microenvironment alterations under clinical treatments (Supplementary Fig. 7). Starfysh successfully identified multiple prostate cancer-enriched niches (hubs shown with dashed lines), along with a unique microenvironment characterized by an abundance of cancer-associated fibroblasts (CAFs; hub 0, pink), which is resistant to androgen-deprivation (AD) therapy. These findings align with those reported by Marklund et al.^30^ and underscore Starfysh’s capability to delineate more specific cell type behavior (Methods). Altogether, these results highlight Starfysh’s ability to derive signal corresponding to structured tissues like the cerebral cortex, pinpoint smaller cells such as tumor-infiltrating immune cells and construct hierarchies of cell types. Such distinctions are impossible with other methods but are crucial for understanding heterogeneous immune responses in healthy and pathological tissues^31^.

### Starfysh dissects the spatial heterogeneity of breast tumors

We further explored the spatial dynamics of immune response in primary breast adenocarcinomas using Starfysh, motivated by heterogeneity in immune cell composition of tumors, which has been linked to variable patient response, for example, to immunotherapy^32–34^. We previously showed that the tissue of residence is a determinant of the diversity of immune phenotypic states and that T cells and myeloid lineage cells exhibit continuous phenotypic expansion in the tumor compared to matched normal breast tissues^35^. Heterogeneous T cell states were defined by combinatorial expression of genes reflecting responses to various microenvironmental stimuli while being tightly associated with T cell receptor (TCR) utilization^35^. These data thus suggested that TCR specificities may contribute to the spatial organization of T cells through the disposition of cognate antigens, facilitating their exposure to niches differing in the extent of inflammation, hypoxia, expression of activating ligands and inhibitory receptors, and nutrient supply.

To investigate this hypothesis, we performed ST profiling of eight primary tumors from an ER^+^ patient, a patient with classic TNBC and two patients with metaplastic TNBC breast cancer (MBC) (two biological replicates each) (Supplementary Table 1 and Methods). The resulting data, alongside published datasets^18^ from a total of six ER^+^ patients and patients with TNBC breast cancer (one biological replicate per patient), were analyzed using Starfysh.

**Fig. 2:**
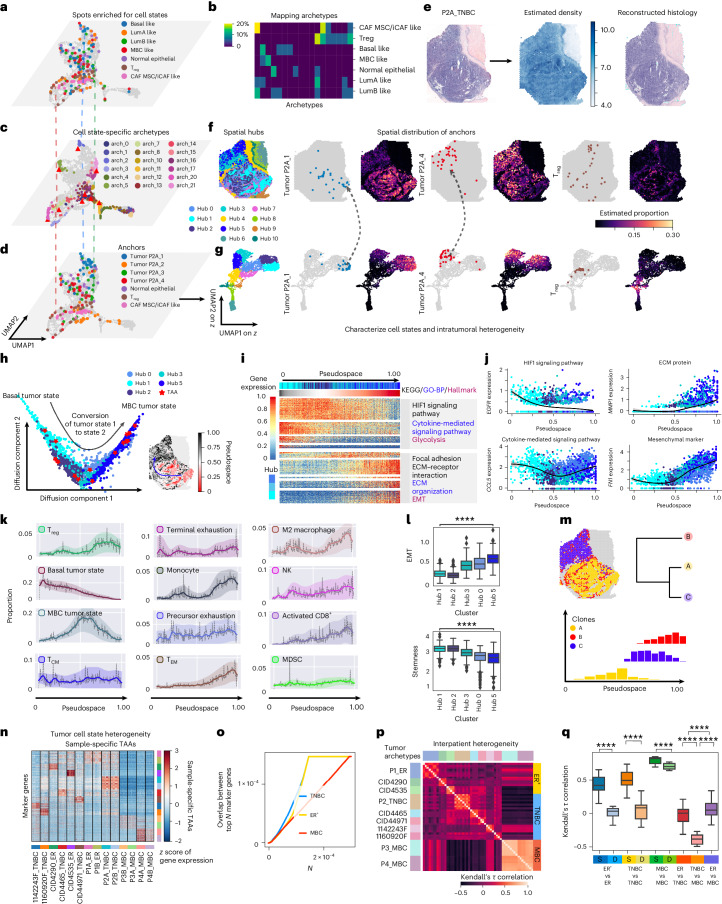
Characterizing spatial tumor heterogeneity in breast carcinoma. **a**, UMAP projection of ST data from the P2A_TNBC sample. Gray dots represent spots; seven example cell states are highlighted in color. See all cell states in Supplementary Fig. 8. MSC, mesenchymal stem cell; iCAF, inflammatory-like cancer-associated fibroblast. **b**, Mapping archetypes to cell states shown in **a**. **c**, Archetypal communities associated with cell states in **a** (Methods). **d**, Spots enriched for cell states are combined with archetypes to achieve a refined anchor set, for example, for patient-specific tumor states. **e**, Histology for sample P2A_TNBC, reconstructed histology and cell density using Starfysh. **f**,**g**, Spatial hubs, distribution of anchors and inferred proportions for two tumor cell states and T_reg_ cells in the spatial context (**f**) and UMAP of Starfysh latent factors (**g**). **h**, Diffusion map analysis of tumor-enriched spots. The dominant trajectory was inferred with SCORPIUS^73^ and is shown in the tissue context (pseudospace axis). **i**, Spatial hubs (top) and pseudospace (middle) for spots sorted along the trajectory inferred in **h**. Heatmaps of expression of gene modules correlated with projections of cells along the trajectory and pathways enriched with gene set enrichment analysis (GSEA; bottom). GO-BP, Gene Ontology Biological Process; KEGG, Kyoto Encyclopedia of Genes and Genomes. **j**, Expression of marker genes in pathways shown in **i** in spots projected on the trajectory. Lines and shading represent local polynomial regression fitting with confidence intervals. **k**, Changes in the proportion of cell states along the pseudospace axis. Data are presented as mean ± s.d. T_CM_, central memory T cell; T_EM_, effector memory T cell. **l**, Expression of gene sets enriched in any intratumoral hub. *n* = 419, 382, 371, 521 and 363 spots were examined. Box plots indicate the median (center lines), interquartile range (hinges) and 1.5× interquartile range (whiskers). One-way ANOVA test was performed across hubs, *P* < 1 × 10^−30^ for EMT and stemness. **m**, Tumor clonality and phylogeny predicted by inferCNV. **n**, Heatmap of expression of the top 20 genes (rows) differentially expressed in TAAs (columns), grouped by sample. **o**, Overlap between the top *N* marker genes differentially expressed in TAAs in any pair of patients. **p**,**q**, Kendall’s *τ* correlation between rankings of genes according to differential expression scores in TAAs (**p**) and grouped by patient subtype (**q**). Correlations among samples from the same (S) and different (D) patients are shown. Box plots indicate the median (center lines), interquartile range (hinges) and 1.5× interquartile range (whiskers). Two-sided independent two-sample *t*-test was performed on Kendall’s *τ* correlations. *P* values = 3.30 × 10^−42^, 5.06 × 10^−48^, 2.01 × 10^−25^, 1.76 × 10^−61^, 5.30 × 10^−66^ and 7.20 × 10^−6^, respectively. ****P < 0.0001, *n* = 96 examined in each subgroup in **q**.

We first dissected the spatial heterogeneity in an individual TNBC tumor and characterized 29 diverse cell states, including normal epithelial, cancer epithelial, immune cells (naive CD4^+^ T cells, effector memory CD4^+^ T cells, myeloid-derived suppressor cells (MDSCs), macrophages, CD8^+^ T cells) and stromal cells (endothelial, perivascular like (PVL), immature PVL). Importantly, given the heterogeneity of tumor cells^36^, Starfysh defined patient-specific tumor cell states by aligning spots enriched for known tumor cell gene sets with archetypes that capture extreme phenotypic states, resulting in refined anchors that guided the deconvolution of spots (Fig. 2a–d and Supplementary Fig. 8). The process of identifying anchors for regulatory T (T_reg_) cells and two tumor cell states is illustrated in Fig. 2a–d, showing an improved separation of cell states after updating gene sets according to archetypes. Additionally, the estimated cell density and the reconstructed image were consistent with the histology (maximal information coefficient = 0.33; compared to 0.18 for shuffled pixels in histology) (Fig. 2e and Methods).

To understand the association between tumor cell phenotypes and the tumor microenvironment (TME), we defined spatial ‘hubs’ as groups of spots with similar composition by applying PhenoGraph^37^ to inferred compositions of spots (Fig. 2f). This analysis revealed that heterogeneous tumor cell states reside in different spatial hubs with more basal-like tumor cells enriched in hub 1, while a second state expressing a subset of MBC-like markers is present in hub 5. These two states correspond to two branches in the inferred latent space (Fig. 2g). This analysis also uncovered regions with varying composition of infiltrating immune cell types exemplified by hub 4 and hub 7 composed of T_reg_-enriched spots (Fig. 2f,g). These results showed Starfysh’s capability to elucidate intratumoral transcriptional heterogeneity and characterize diverse and patient-specific tumor cell states, in part determined by their spatial context and colocalization with immune subsets.

### Starfysh shows a spatially covarying tumor–immune transition

Further analysis of spots enriched for tumor cells using diffusion maps^38,39^ revealed a continuous transition from basal to MBC-like tumor cell states corresponding to a spatial gradient (Fig. 2h and Supplementary Fig. 9a). The inferred trajectory (pseudospace axis) is associated with upregulation of extracellular matrix (ECM) organization and ECM–receptor interaction pathways and loss of cytokine-mediated signaling-related gene expression, and glycolysis (Fig. 2i,j). The upregulation of epithelial–mesenchymal transition (EMT)-related and collagen genes, which are associated with metastatic potential^40–42^, as a gradient reproduced in the adjacent tissue sample re-enforces the concept that intratumoral heterogeneity is a continuum rather than abruptly demarcated cell states. Indeed, projecting all anchors enriched for tumor gene sets as ‘tumor-associated anchors’ (TAAs) showed that they are uniformly distributed along the pseudospace axis (Fig. 2h), representing different stages of this transformation.

We then sought to investigate whether different immune cell states are associated with regions with varying tumor phenotypes. Remarkably, we found a compositional shift from central memory and precursor exhausted T cell states^43^ to effector memory, terminally exhausted and T_reg_ states, as colocalized tumor cells lose basal properties along the pseudospace axis, while activated T cells are observed at the tumor margins (Fig. 2k). These observations indeed suggest that different T cell states are associated with various niches of the TME shaped by varying nutrient supply, oncogenic signals and tumor cell differentiation states. In parallel, tissue-repair (M2) macrophages, which have been implicated in promoting invasion, migration and proliferation of TNBC cells^44^, were elevated toward the periphery.

The tumor state transformation axis coincides with a loss of stemness, a gain in EMT and downregulation of WNT signaling gene sets (Fig. 2l and Supplementary Fig. 9b,c). Examining tumor clonality by applying inferCNV^45^ suggests distinct copy number profiles associated with basal and mesenchymal-like phenotypic states residing in different locations (Fig. 2m and Supplementary Fig. 9d). To further investigate tumor–immune colocalization, we adopted a TCR amplification protocol^46^ in an MBC tumor (P4A_MBC), identifying a dominant T cell clone spatially distributed across the tissue (Supplementary Fig. 10a–d). Deconvolved cell states from Starfysh suggest that spots associated with this clonotype varied in T_reg_ cell and precursor exhausted T cell proportions, determined by their location (Supplementary Fig. 10e,f). This result accords with other studies on conversion of naive CD4^+^ T cell clones into T_reg_ cells^47^ and T_reg_ cells implicated in promoting T cell exhaustion^48^.

In addition to characterizing intratumoral heterogeneity, Starfysh also quantifies intertumor heterogeneity. By performing differential gene expression analysis, we identified markers characterizing TAAs in all breast tumor samples. Marker gene sets for tumor states in biological replicates originating from the same patient tumor were overlapping as expected, while distinct modules of non-overlapping markers illustrate intrapatient heterogeneity (Fig. 2n). Quantifying the overlap in top marker genes of tumor states across patients of the same subtype, we observed greater divergence in markers representing MBC tumor states, implicating higher intertumoral heterogeneity in MBC samples than that in TNBC and ER^+^ samples (Fig. 2o), consistent with the known morphological heterogeneity of MBCs^49^. The heterogeneity between TNBC and MBC was further supported by comparing rankings of TAA differentially expressed genes, where we found a lower correlation between patients with MBC and TNBC than in samples of the same subtype (Fig. 2p,q).

### Starfysh defines spatial hubs from integrated breast tumors

**Fig. 3:**
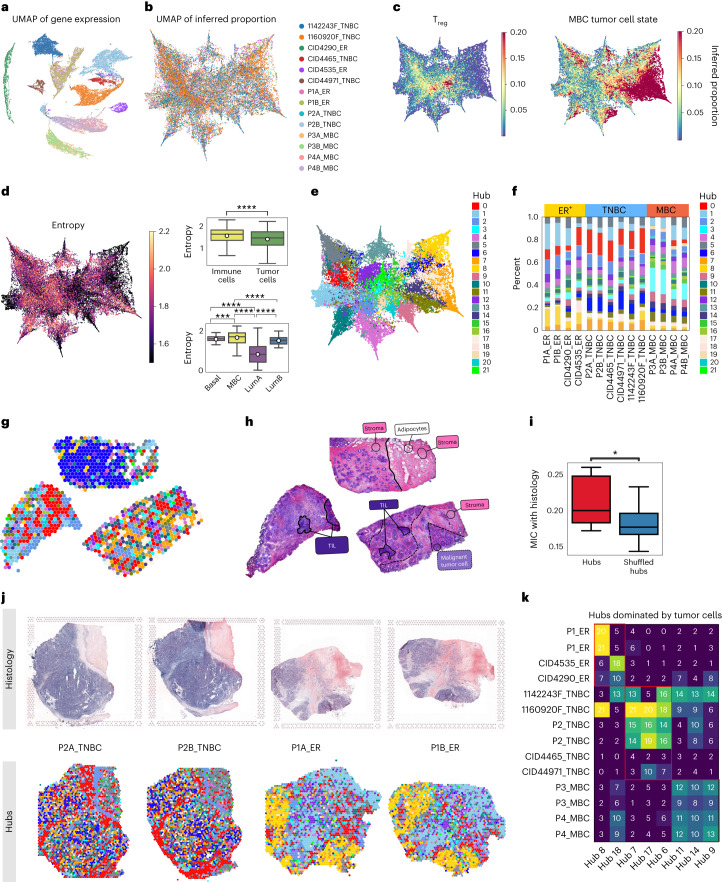
Characterizing tumor–immune hubs from the integration of samples. **a**,**b**, UMAP visualization of ST data from four MBC, six TNBC and four ER^+^ samples (*n* = 37,517 spots) before (**a**) and after (**b**) Starfysh integration on the joint latent space of **c**. **c**, UMAP visualization of Starfysh-inferred proportions from integration of spots from all samples colored by the proportions of a tumor cell state and an example immune cell state (T_reg_) in the integrated space. **d**, UMAP of integrated space colored by Shannon’s entropy per spot and box plots of entropy, grouping spots by disease subtype. Box plots indicate the median (center lines), interquantile range (hinges) and 1.5× interquartile range (whiskers). *n* = 32,409 immune cell-enriched spots and 5,108 tumor cell-enriched spots. *n* = 47, 493, 467 and 74 in basal-, MBC-, LumA- and LumB-enriched spots. Two-sided independent two-sample *t*-test was performed on the entropy of each group comparison. *P* value = 7.89 × 10^−160^ in comparison between immune cells and tumor cells; *P* values = 1.08 × 10^−2^, 2.04 × 10^−142^, 2.30 × 10^−52^, 1.99 × 10^−49^, 2.31 × 10^−7^ and 2.14 × 10^−2^ for basal versus MBC, MBC versus LumA, LumA versus LumB, basal versus LumA, MBC versus LumB and basal versus LumB. ***P < 0.001, ****P < 0.0001. **e**, UMAP of integrated space colored by hubs identified by clustering spots based on inferred cell type proportions. **f**, Spatial hub distribution for each sample. **g**,**h**, Spatial arrangement of hubs (**g**) and pathological histology annotation of sample 44971_TNBC (**h**). Inferred hubs align well with annotated DCIS (red hub), lymphocyte-infiltrated (olive green hub) and stroma (yellow hub) regions. TIL, tumor-infiltrating lymphocyte. **i**, MIC for alignment of hubs with histology. Box plots indicate the median (center lines), interquantile range (hinges) and 1.5× interquartile range (whiskers). *n* = 1,162 spots in both hubs and shuffled hubs. Two-sided independent two-sample *t*-test was performed. *P* value = 1.30 × 10^−2^. *P < 0.05. **j**, Paired histology and spatial arrangement of hubs for TNBC and ER^+^ patient samples showing consistencies between replicates of the same patients and with histology. **k**, Number of spots assigned to intratumoral hubs in each patient.

To demonstrate the potential of Starfysh in deriving commonalities among heterogeneous samples and disease subtypes, we performed an integrated analysis of all 14 samples from ten patients (*n* = 37,517 spots) (Supplementary Table 3 and Methods). Uniform manifold approximation and projection (UMAP) dimensionality reduction of ST data without Starfysh revealed no overlap among patients, partly due to patient-to-patient variation, given that replicate samples overlapped (Fig. 3a). Moreover, the aggregation of patient-specific tumor cells with other cell types within spots hindered the comparison of shared immune states and spatial neighborhoods between patients. While batch correction methods designed for single-cell data failed in correcting the variations between patients (Supplementary Fig. 11a,b), Starfysh successfully integrated all datasets in a joint latent space (Fig. 3b and Supplementary Figs. 11c and 12). It yielded greater mixing of immune states quantified with the entropy of the local distribution of patients (Methods) yet preserved differences between patient-specific tumor cells (Fig. 3c,d). Overall, this analysis showed that MBC tumors have the highest heterogeneity, while luminal (Lum)A tumors display lower heterogeneity than other subtypes.

To understand similarities and differences in the organization of cell states among patients, we identified spatial hubs from the integration of all samples (Fig. 3e). The majority of hubs were detected in more than one patient (Fig. 3f). The distribution of hubs, however, varied between disease subtypes and patients. The spatial arrangement of hubs showed a marked similarity to expert-annotated histology, including in rare normal epithelium regions, tumor-infiltrated regions and immune cell-enriched regions (Fig. 3g,h), which was quantified using the maximum information coefficient (MIC) (Fig. 3i and Methods). As expected, hub distributions had similar patterns between replicates, that is, adjacent sections of tumor tissues (for example, P1A_ER, P1B_ER), whereas hubs dominated by tumor cells were different between patients (for example, P1, P2) (Fig. 3j,k).

### Hypoxia shapes an immunosuppressive niche in MBC

By integrating ST datasets, we systematically compared tumor heterogeneity and its interplay with tumor–immune characteristics across breast cancer subtypes. In particular, we investigated potential differences in cellular organization in MBC compared to other TNBCs^50^. MBC is a rare and aggressive form making up 1–2% of all breast cancer^40^ and typically characterized as TNBC due to lack of expression of ER, progesterone receptor (PR) and human epidermal growth factor 2 receptor (HER2). However, MBCs have worse prognosis and greater resistance to chemotherapy than conventional TNBC^40,51,52^. A hallmark of MBC is morphological heterogeneity, reflected in its name^49,53^. This distinguishing feature alongside enrichment in macrophages and immunosuppressive T_reg_ cells^54^ motivates the spatial characterization of tumor–immune crosstalk in the MBC TME to help guide the development of new therapeutic approaches tailored to MBC’s unique biology.

**Fig. 4:**
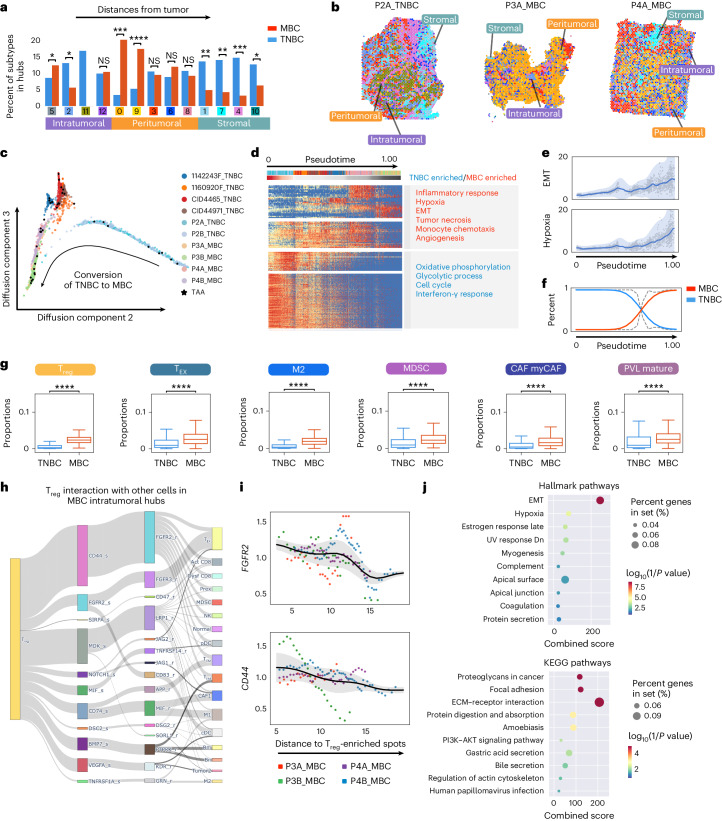
Intratumoral inflammation and heterogeneity in MBC epithelia. **a**, Classification of spatial hubs according to distance from tumor hubs and matched histology. Percentage of spots from MBC and TNBC subtypes in each hub. One-sided independent two-sample *t*-test was performed for comparisons of proportions in each hub. *P* values = 3.05 × 10^−2^, 1.48 × 10^−2^, 0.43, 2.74 × 10^−4^, 9.13 × 10^−5^, 0.63, 0.94, 0.77, 3.80 × 10^−3^, 4.65 × 10^−3^, 1.05 × 10^−4^ and 3.84 × 10^−2^, sequentially. *P < 0.05, **P < 0.01, ***P < 0.001, ****P < 0.0001. NS, not significant. **b**, The spatial arrangement of hubs. **c**, Diffusion map analysis reveals a continuous trajectory between TAAs across different MBC and TNBC patient samples. Archetypes are shown, with black stars representing the most distinct states for TAAs. The dominant trajectory was inferred with SCORPIUS^73^. **d**, Top row: spots ordered by inferred pseudotime using SCORPIUS based on diffusion components in **c**. Second row: pseudotime for spots sorted along the trajectory inferred in **c**. Bottom: heatmaps of expression of gene modules with positive or negative correlation with the projection of cells along the trajectory and select pathways enriched with GSEA. **e**, Expression of EMT- and hypoxia-relevant gene sets shows highly correlated dynamics along pseudotime. Data are presented as mean values ± s.d. **f**, Percentage of TNBC and MBC spots along the inferred pseudotime. **g**, Comparison of inferred intratumoral cell state proportions across tumor subtypes. T_EX_, terminal exhausted T cells; myCAF, myofibroblast-like cancer-associated fibroblasts. Box plots indicate the median (center lines), interquantile range (hinges) and 1.5× interquartile range (whiskers). *n* = 5,366 and 1,888 intratumoral spots for TNBC and MBC, respectively. Two-sided independent two-sample *t*-test was performed. *P* < 1 × 10^−30^, 1.20 × 10^−38^, 4.21 × 10^−220^, 8.06 × 10^−30^, 4.80 × 10^−68^ and 3.26 × 10^−17^, sequentially. **h**, Predicted significant receptor–ligand interactions between T_reg_ cells (sender) and other cell types (receiver) in MBC intratumoral regions. Prex, precursor exhausted T cells; pDC, plasmacytoid dendritic cells; cDC, conventional dendritic cells; Bm, memory B cells; Bn, naive B cells. **i**, *FGFR2* and *CD44* expression averaged across spots in each tumor subtype after binning according to *k*-nearest neighbors (kNN) graph path length from T_reg_-enriched spots in intratumoral hubs. Data are presented as mean values ± s.d. **j**, Enrichment analysis for MBC intratumoral hubs. Differentially expressed genes were identified using the Wilcoxon test in Scanpy, and significant pathways (false discovery rate < 0.05, Benjamini–Hochberg) are shown with GSEA’s default permutation-based test. UV, ultraviolet. Dn, downregulated.

In our comparative analysis of TNBC and MBC tumors, we defined spatial hubs among ten samples encompassing these subtypes (Supplementary Fig. 13a and Methods) and partitioned them into intratumoral, peritumoral and stromal categories according to spatial arrangement around tumor regions (Fig. 4a and Supplementary Fig. 13b–d). Distinct intratumoral hubs across samples highlight tumor cell heterogeneity among patients (for example, hub 11; Figs. 3k and 4a,b). To understand phenotypic differences in MBC tumor states, we projected TAAs onto the inferred joint space from integration of all samples (Methods) and applied diffusion map analysis. This revealed tumor state transition trajectory from a TNBC-enriched state to an MBC-specific state correlated with tumor growth regulation and reduced glycolytic processes (Fig. 4c,d). MBC-specific states were associated with inflammatory response, hypoxia, EMT and tumor necrosis. The expression of EMT- and hypoxia-related genes, along with sample distribution on this trajectory confirmed their enrichment in MBC intratumoral hubs (Fig. 4e,f). Oncogenic pathways like PI3K–AKT, anti-inflammatory and glucose-deprivation pathways were enriched in MBC intratumoral hubs, while G2/M and pro-inflammatory pathways were downregulated (Supplementary Fig. 13e), suggesting an immunosuppressive environment in MBC intratumoral regions.

In parallel, we observed an increase in hypoxia approaching MBC intratumoral hubs, accompanied by enrichment in T_reg_ and PVL cells in MBC (Fig. 4d–g). In fact, enrichment of T_reg_ cells colocalizing with exhausted T cells (as determined by the spatial correlation index^55^) in intratumoral hubs was detected only in MBC (Supplementary Fig. 14a and Methods), implicating T_reg_ infiltration as a potential hallmark of MBC.

To identify communication patterns used by MBC tumor-infiltrating T_reg_ cells, we predicted receptor–ligand interactions that may mediate crosstalk between T_reg_ cells and other cell states in intratumoral hubs using CellPhoneDB^56^ (Fig. 4h, Supplementary Fig. 14b,c and Methods), revealing immunosuppressive pathways related to *FGF2*, *FGFR1* and *CD44* expression involved in MBC. Notably, FGF2 is a protumor angiogenesis factor and induces drug resistance in chemotherapy in breast cancer^57^. The receptor FGFR1 induces the recruitment of macrophages and MDSCs in the tumor^58^, while CD44 is a known marker of breast cancer stem-like cells and stabilizes T_reg_ persistence and function^59^. We observe diffused expression of these receptors with distance from T_reg_-enriched spots in MBC (Fig. 4i), further supporting their involvement in intratumoral T_reg_ communication. These results demonstrate complex crosstalk in response to the immunosuppressive signals generated by T_reg_ cells.

Aside from T_reg_ cells, other immunosuppressive cells such as M2-like macrophages, MDSCs and CAFs were also uniquely enriched in MBC intratumoral hubs compared to TNBC ones (Fig. 4g). Previous studies have shown that hypoxia affects EMT in cancer by regulating EMT signaling pathways, EMT-associated microRNA and long noncoding RNA networks^60^. Both hypoxia and EMT were reported to modulate the TME by recruiting immunosuppressive cell types such as T_reg_ cells^61,62^, in line with our observation (Fig. 4g), implicating hypoxia as a major factor contributing to MBC. Hypoxia is also known to confer therapy resistance by inducing cell cycle arrest and inhibiting apoptosis and mitochondrial activity^63^. Therefore, a tumor subpopulation surviving hypoxia may contribute to resistance to chemotherapy and radiotherapy.

Gene enrichment analysis in MBC intratumoral hubs consistently revealed EMT, hypoxia, ECM and PI3K–AKT signaling in MBC samples (Fig. 4j and Supplementary Fig. 14d,e). Notably, the genomic landscape of MBCs shows frequent mutations in *TP53* and the PI3K–AKT–mammalian target of rapamycin (mTOR) pathway^64,65^. Our data thus suggest possible coordination of nutrient uptake including glucose through hypoxia-inducible factor 1 (HIF1) and PI3K–AKT pathways^66^, supporting enhanced growth and proliferation in intratumoral MBC hubs^67^, while this metabolic reprogramming is associated with immunosuppressive crosstalk.

### Spatial organization and interactions in the stromal breast TME

**Fig. 5:**
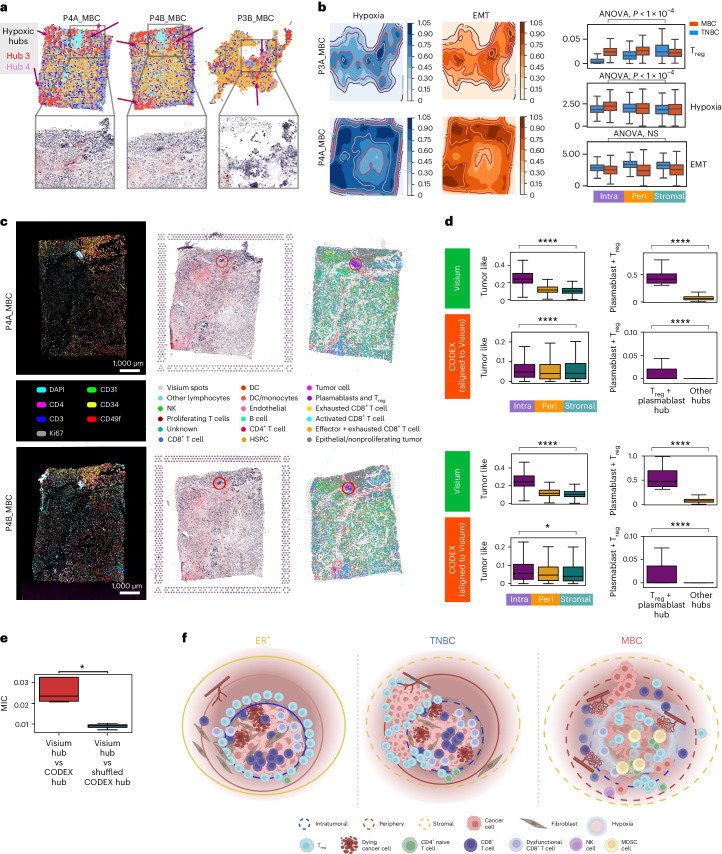
Spatial heterogeneity of the stromal breast TME. **a**, Spatial arrangement of hubs and corresponding histology indicate blood cells and vessels around hypoxic hubs (hubs 3 and 4) in MBC. **b**, Contour map and bar plots showing expression gradients of EMT- and hypoxia-related gene sets. Top: sample P3A_MBC; bottom: sample P4A_MBC. One-way ANOVA test was performed on box plots of inferred T_reg_ proportions and expression of EMT- and hypoxia-related gene sets for regions in MBC. *P* values = 1.46 × 10^−29^, 1.04 × 10^−36^ and 0.12, respectively. Box plots indicate the median (center lines), interquantile range (hinges) and 1.5× interquartile range (whiskers). *n* = 5,366 and 1,888 spots in intratumoral regions, 5,608 and 7,104 spots in peritumoral regions, and 7,524 and 1,463 spots in stromal regions for TNBC and MBC, respectively. Intra, intratumoral; Peri, peritumoral. **c**, A subset of CODEX markers, histology and segmented single cells from CODEX images aligned with Visium for sample P4A_MBC and sample P4B_MBC. DAPI, 4,6-diamidino-2-phenylindole; DC, dendritic cell; HSPC, hematopoietic stem and progenitor cell. **d**, Comparisons of tumor and plasmablast–T_reg_ percentages between inferred results in Visium and aligned CODEX in intratumoral, peritumoral and stromal hubs. *n* = 584, 1,863 and 652 spots in intratumoral, peritumoral and stromal hubs; *n* = 83 and 3,090 spots in the T_reg_–plasmablast hub and other hubs. A one-way ANOVA test across regions was performed. *P* value = 0 for all Visium-related box plots, *P* values = 6.25 × 10^−5^ and 0.03 in tumor-like proportions in P4A_MBC and P4B_MBC samples, and *P* values = 5.06 × 10^−57^ and 0 in plasmablast and T_reg_ cell proportions in P4A_MBC and P4B_MBC samples. Box plots indicate the median (center lines), interquantile range (hinges) and 1.5× interquartile range (whiskers). **e**, MIC between hubs identified from Visium and hubs found in CODEX. *n* = 4 samples for Visium and CODEX, respectively. A one-sided independent two-sample *t*-test was performed. *P* value = 1.67 × 10^−2^. Box plots indicate the median (center lines), interquantile range (hinges) and 1.5× interquartile range (whiskers). An ANOVA test was performed for comparisons. *P < 0.05, ****P < 0.0001. **f**, Summary diagram.

To dissect the stromal TME responding to unique microenvironment niches, such as gradients of hypoxia in MBC, we characterized the cellular composition of peritumoral and stromal regions (Fig. 4a). Intriguingly, T_reg_-enriched hubs 3 and 4 were present in all samples but showed unique patterns in each disease subtype (Supplementary Fig. 13f). For example, they enveloped tumor hubs or were spatially scattered in TNBC tumors (Fig. 4a,b; for example, hubs 3 and 4 in P2A_TNBC). This feature of tumor hubs enveloped with T_reg_-enriched regions was also identified in ER^+^ tumor samples (P1A_ER, P1B_ER in Fig. 3j with T_reg_-enriched hubs 0 and 2). By contrast, in MBC, they were concentrated at certain locations close to intratumoral hubs (Fig. 5a and Supplementary Fig. 12). In addition to the spatial shifts of T cell states, endothelial cells (CAFs; Fig. 4g) were also enriched in hubs 3 and 4 in MBC, suggestive of heightened angiogenesis in the stromal TME of MBC, which was particularly apparent in histology of the region, likely as an adaptation to hypoxia (Fig. 5a,b).

To validate Starfysh’s predictions, we performed co-detection-by-indexing (CODEX) profiling on MBC tissues with 23 antibodies (Supplementary Fig. 15a–d and Supplementary Table 6). As a multiplexed imaging technology, CODEX measures single-cell protein expression. The profiled tissues were resectioned adjacent to those profiled with ST and showed similar tissue architecture in histology. Aligning the segmented and annotated single-cell CODEX data with ST data confirmed the predicted spatial organization of major and rare cell types. For example, CODEX-profiled regions enriched for T_reg_ cells and plasmablasts aligned with hub 7 in ST samples, adjacent to the intratumoral regions (Figs. 5c and 4a,b and Supplementary Fig. 15e). The cellular components of vasculature indicated by CD31 expression also matched predicted endothelial and perivascular cells in ST data. We further assembled the single-cell CODEX into spot-level resolution and compared proportions of cells across TME regions. We identified a decline in tumor cells from intratumoral to stromal regions and a unique enrichment of T_reg_ cells and plasmablasts at the tumor border (Fig. 5d). We then compared cell neighborhoods defined according to CODEX to spatial hubs in ST and found a significant correlation (Fig. 5e and Methods). Overall, Starfysh enabled characterization of the spatial TME in MBC differing from TNBC and ER^+^ cancer (summarized in Fig. 5f). Our analysis suggests that the enriched tumor-suppressive cells in MBC intratumoral regions underlying heightened hypoxia and EMT potential and angiogenesis in the MBC TME likely oppose pro-inflammatory responses and limit CD8^+^ T cell infiltration (Supplementary Fig. 15f).

## Discussion

By incorporating archetypal analysis and prior knowledge of cell state markers in a deep generative model, Starfysh dissects the spatial heterogeneity of complex tissues from ST and histology, without relying on single-cell references. It refines cell states using archetypes and deconvolves them using a generative model enhanced with histological data, providing information on tissue architecture, cell density and spatial dependencies between measurements. Starfysh excels in integrating multiple heterogeneous tissue samples and identifying shared or tissue-specific cell states and spatial hubs. These key features make Starfysh an ideal tool to discover spatial hubs from integrated large-scale datasets, increasing power to detect features of complex and rare diseases that could drive future therapeutic strategies.

Applied to breast tumors, Starfysh elucidated the role of spatial heterogeneity in shaping continuous phenotypic expansion of tumor-infiltrating immune cells^35^. It revealed a correlation between tumor cell state transitions and immune cell distribution, supporting the hypothesis that tumor cell spatial orientation influences immune differentiation.

We demonstrate the power of Starfysh in integrating multiple tissues using our generated and previously published ST datasets. This integration allowed for quantification of intratumoral and intertumoral heterogeneity and identification of spatial hubs with similar cell state compositions. A key application of this integration was comparing rare, chemoresistant metaplastic breast tumors to other breast cancer subtypes. Notably, we found intratumoral infiltration of T_reg_ cells, M2-like macrophages and MDSCs in MBC, shaping an immunosuppressive niche enriched in EMT and hypoxia. Crosstalk with T_reg_ cells was predicted to be mediated through FGF2, FGFR1 and CD44 signaling pathways, which would be top candidates for future functional studies. Indeed, FGFR signaling is known to maintain EMT-mediated drug-resistant populations^68^. Enrichment of p53 and PI3K–AKT pathways in MBCs also suggests reprogramming of metabolic activity in MBC tumors. Our data thus motivate further investigation of FGFR inhibitors^69^ as well as other approaches for targeting glucose metabolism^70^ and immunosuppressive T_reg_ cells for the treatment of MBCs.

In addition to spatial characterization of the TME specific to this rare subtype of breast cancer, the integration identified a stromal hub shared across breast cancer subtypes while exhibiting varying spatial patterns. Within this stromal hub, we observed compositional shifts with the replacement of T_reg_ cells with activated CD8^+^ T cells in MBC compared to other TNBCs. Additionally, our observation of enriched endothelial cells in MBC stroma alludes to mechanisms of local adaptation to hypoxic regions through possible vascular formation. Altogether, these results imply that the underlying biology of the tumor impacts stromal response and immune infiltration.

Overall, Starfysh has proven effective in analyzing complex ST, integrating patient samples with distinct microenvironments and sources, and has demonstrated robustness in characterizing spatial interactions within and across samples. These features enabled extraction of biological insights from a limited cohort of patients with breast cancer. In a recent study, we applied Starfysh to disentangle the spatial dynamics of activated and exhausted T cell subsets in Slide-seqV2 (ref. ^71^) data from anti-PD-1-treated melanoma tumors^72^, showing its applicability to other ST technologies and cancer systems. In future work, incorporation of archetypal analysis in the probabilistic framework and extensions to multiomic integration with proteomics or chromatin accessibility will improve our ability to achieve comprehensive characterization of spatial heterogeneity. Additionally, integration with high-resolution images can explicitly account for cell morphology.

## Methods

### Starfysh model

#### Model overview

Deep generative models parameterized by neural networks have proven effective in analyzing single-cell RNA expression data (scvi-tools^19^, scVI^20^, totalVI^21^, scArches^22^, trVAE^23^, scANVI^24^, MrVI^25^ and so on). However, the presence of multiple cell types in each spot in ST data makes it difficult for these models to disentangle cell type-specific features. To overcome this limitation, Starfysh introduces a generative model with a special variational family that is structured to model the presence of multiple cell states per spot in ST data. The Starfysh generative model leverages gene set signatures (either existing signatures or signatures computed with archetypal analysis) as an empirical prior to help disentangle cell types^72^. We first detail the generative model of Starfysh and then introduce its structured variational family.

#### Starfysh generative process

Starfysh models the vectors of gene expression ${x}_{i}\in {{\mathbb{R}}}^{G}$ (with *G* the number of observed genes) for each spot *i* with a generative model. The generative model (Fig. 1c) is parameterized by *K*, representing the expected number of cell states in the data. The determination of *K* can be automated through archetypal analysis beforehand, or an expert can provide guidance on the *K* most important cell states in the sample. Each cell state *k* ∈ [*K*] is characterized by a low-dimensional latent variable, ${u}_{k}\in {{\mathbb{R}}}^{D}$ (with *D* defaulting to ten dimensions), capturing the specific mechanisms underlying that cell state. Moreover, each cell state *k* has a scalar variable, *σ*_*k*_ > 0, indicating its variability and heterogeneity.

Subsequently, Starfysh models each spot *i* with a specific low-dimensional representation *z*_*i*_. In the context of single-cell data, each cell state *k* would usually be represented by a low-dimensional vector *z* centered around *u*_*k*_, with a standard deviation of *σ*_*k*_. However, for ST data, where each spot captures a mixture of cells with different cell states, Starfysh associates each spot *i* with a proportion vector, *c*_*k*_ ∈ Δ^*K*^, representing the proportions of each cell state in that spot. Starfysh then constructs the low-dimensional representation *z*_*i*_ with a mixture distribution that combines the cell state proportions *c*_*i*_ and the cell state-specific representations *u*_*k*_: ${z}_{i}|{c}_{i},{u;}\,\sigma \sim N({\sum }_{k}{c}_{{ik}}{u}_{k},{\sum }_{k}{c}_{{ik}}{\sigma }_{k})$.

Following this, *z*_*i*_ is transformed using a neural network *f* to obtain the normalized mean expression of each gene for spot *i*, which is further scaled by the library size *l*_*i*_. The observed raw transcript count *x*_*ig*_ for gene *g* in spot *i* is then sampled from a negative binomial distribution centered around the upscaled mean.

Cell state proportions, *c*_*i*_, are also considered as random variables with a carefully crafted prior. Each cell state *k* ∈ [*K*] needs to be associated with a preliminary gene set signature, *s*_*k*_, which can be provided by the user or automatically discovered through archetypal analysis. By calculating the signature scores in each spot, denoted as *A*(*x*_*i*_, *s*_*k*_), Starfysh establishes a prior distribution over the cell state proportions in each spot. Specifically, the proportions of cell states *c*_*i*_ are sampled from a Dirichlet distribution with a prior parameter *α*[*A*(*x*_*i*_, *s*_*k*_)]_*k∈*[*K*]_. For instance, if spot *i* highly expresses known marker genes for cell state *k*, then a larger value of *A*(*x*_*i*_, *s*_*k*_) will favor the probability of allocating cell state *k* for spot *i* according to the empirical Dirichlet prior parameter. The parameter *α* modulates the prior strength and represents the belief in the signature gene sets: a larger value corresponds to a stronger prior, while a smaller value results in a less constraining prior.

The generative model is defined as $$p(u,{c},{z},{l},{x})={\prod }_{k=1}^{K}p({u}_{k}){\prod }_{i=1}^{n}$$$$p({c}_{i})p({z}_{i}|{c}_{i},u)p({l}_{i})p({x}_{i}|{z}_{i},{l}_{i})$$, with

- *p*(*u*_*k*_) = Normal (0, 10*I*_*D*_)
- *p*(*c*_*i*_; *α*, *A*) = Dirichlet (*α*⋅*A*), where *α* controls the prior strength on the signature scores *A*.
- *p*(*z*_*i*_|*c*_*i*_, *u*; *σ*) = ${\rm{Normal}}(\sum _{k}{c}_{{ik}}{u}_{k},\sum _{k}{c}_{{ik}}{\sigma }_{k})$, where the parameters *σ*_*k*_ represent cell state-specific heterogeneity.
- $p({l}_{i}{\rm{;}}\widetilde{{l}_{i}})={\rm{logNormal}}(\widetilde{{l}_{i}},1)$, where $\widetilde{{l}_{i}}$ is the locally averaged library size observed in spot *i*’s spatial neighborhood.
- *p*(*x*_*i*_|*z*_*i*_, *l*_*i*_) = $${\prod }_{g=1}^{G}p\left({x}_{{ig}}{\rm{|}}{l}_{i},{z}_{i}\right)\,$$
- *p*(*x*_*ig*_|*l*_*i*_, *z*_*i*_; *θ*_*g*_, *f*) = NegativeBinomial (*l*_*i*_*f*(*z*_*i*_), *θ*_*g*_), where *θ*_*g*_ denotes gene-specific dispersions and *f* is a neural network with a softmax output.

In the generative process, the parameters $A,\alpha ,\widetilde{{l}_{i}}$ are fixed. The prior strength *α* is set by default to 50. Robustness analysis on *α* demonstrates that the model consistently outperforms the signature prior given a reasonable range (*α* ≥ 1) (Supplementary Fig. 2c). The optimal choice of the prior strength term depends on the specific dataset and markers. The locally averaged library size is computed as $\widetilde{{l}_{i}}=\frac{1}{|{N}_{i}|}\,\sum _{j\in {N}_{i}}{\sum }_{g}\;{x}_{{jg}}$, where *N*_*i*_ is the set of spots physically located adjacent to spot *i* and also includes *i*. The cell state heterogeneities *σ*_*k*_ are initialized as 1, and the gene dispersions *θ*_*g*_ are initialized at random. Finally, the neural network *f* has by default one linear layer followed by a softmax. *σ*_*k*_, *θ*_*g*_ and *f* are all learned during the inference.

#### Integration with histology images

Although histology hematoxylin-and-eosin (H&E) images are usually provided along with ST data (for example, the commercial Visium platform), current methods fail to use such modality in deconvolving cell types. Histology, however, provides useful information about morphology, tissue structure, cell density and spatial dependency of cells. Integrating histology and transcriptomes in a joint model is challenging, as the two data modalities are very different: the genome-level transcripts are high-dimensional vectors, whereas the histology data consist of multichannel images. Thus, it is essential to address the mismatch of these two types of data while preserving cell type-specific information of gene expression and cell morphology-specific information of histology images. The integrative approach in Starfysh is formulated with a deep variational information bottleneck^26^.

The original H&E images are first normalized to [0, 1] per channel. The alignment between H&E images and ST spot *i* produces the histology image patches ${y}_{i}\in {{\mathbb{R}}}^{P\times P\times C}$ (with *P* as the side length of the patch and *C* as the number of image channels, for example, *C* = 3 for RGB images and *C* = 1 for grayscale images). We set *P* = 26 by default to approximate the number of pixels surrounding each spot. The image patch *y*_*i*_ is then flattened in the Starfysh model and assumed to be generated from the same latent variable *z*_*i*_ that informs gene expression (Fig. 1c and Supplementary Fig. 1a) with a distribution *p*(*y*_*i*_|*z*_*i*_) parameterized by two neural networks *g*_*μ*_, *g*_*σ*_, for mean and variance of distribution for *y*_*i*_, respectively. Both consist of a linear layer followed by a batch normalization layer. They define:

$$p\left(\;{y}_{i}{\rm{|}}{z}_{i}\right)={\rm{Normal}}\left(\;{g}_{\mu }({z}_{i}),{g}_{\sigma }({z}_{i})\right).$$

#### Construction of the empirical prior

For cell states expected to reside in the tissue, Starfysh first filters out marker genes that are either unavailable in the ST data or not expressed in any spots to obtain binary variable ${s}_{k}\in {{\mathbb{R}}}^{G}$, *k* = {1,…, *K*}. Next, two priors are calculated before running Starfysh, including a prior for the cell state proportions that reflects their spot enrichment and a prior for the library size:

1. Prior for the cell type proportion:
  *A*(*x*_*i*_, *s*_*k*_) is defined as the enrichment score^74^ of the marker genes for cell state *k* at spot *i*. The score is first calculated with the Scanpy function ‘scanpy.tl.score_genes’, which computes the marker genes’ average expression and subtracts from it the average expression of a reference gene set *G*′ randomly sampled from binned expressions: ${A}^{{\rm{raw}}}({x}_{i},{s}_{k})=\frac{1}{|{s}_{k}{|}}{\sum }_{g\in G}\;{x}_{{ig}}\cdot {s}_{{kg}}-\frac{1}{{|G^\prime|}}{\sum }_{g\in {G^\prime}}{x}_{{ig}}$. We further transformed the scores using the function ReLU(*x*) = max(0, *x*) to ensure the positive constraints of Dirichlet parameters and make them comparable across spots (with *ϵ* defaulting as 1 × 10^−5^):
  $$A({x}_{i},{s}_{k})={\rm{ReLU}}({A}^{{\rm{raw}}}({x}_{i},{s}_{k}))+\epsilon$$
  $$A({x}_{i},{s}_{k})=\frac{A({x}_{i},{s}_{k})}{{\varSigma }_{k}A({x}_{i},{s}_{k})}.$$
  For each cell state, the prior assigns unique enrichment scores across all spots, and we thus can define the anchor spots $R\in {{\mathbb{R}}}^{S\times K}$ specifying the ranking of each spot *i* based the enrichment score $A(:,{k})$ for each state $k$, which can be updated with archetypal analysis detailed below.
2. Prior for the library size:
  Starfysh also considers the spatial dependency of spots when generating the prior for library size. $\widetilde{{l}_{i}}=\frac{1}{|{N}_{i}|}\,\sum _{j\in {N}_{i}}{\sum }_{g\,}{x}_{{jg}}$, where ${N}_{i}$ is the set of spots physically located around the spot *i*, which includes all spots *j* such that $|{r}_{{j}}-{r}_{i}| < w$, where *w* is an adjustable parameter for window size (default set to 3). ${r}_{i}$ is the spatial coordinates for spot *i*.

#### Archetypal analysis

Marker genes that represent cell states may be context dependent or unknown. To address these limitations and improve the characterization of tissue-dependent cell states, we developed a geometric preprocessing step, leveraging archetypal analysis^75^, to refine marker genes and identify new cell states.

Archetypal analysis fits a convex polytope to the observed data, finding the prototypes (archetypes) that are most adjacent to the extrema of the data manifold in high dimension. Previous works^76–78^ have applied archetypal analysis to scRNA-seq data to characterize meaningful cell types. In the context of ST, we hypothesize that the archetypes are closest to the purest spots that contain only one or the fewest number of cell states, while the rest of the spots are modeled as the mixture of the archetypes.

We applied the PCHA algorithm^79^ to find archetypes that best approximate the ‘extrema’ spots on a low-dimensional manifold. Specifically, let $\hat{X}\in {{\mathbb{R}}}^{S\times G}$ be the normalized spot (*S*) by gene (*G*) expression from the original spatial count matrix. We further selected the first *P* = 30 principal components (${X^\prime}\in {{\mathbb{R}}}^{S\times P}\,$) to denoise the data. We denote matrices $W\in {{\mathbb{R}}}^{S\times D},{B}\in {{\mathbb{R}}}^{D\times S}$ and $H={BX^\prime}\in {{\mathbb{R}}}^{D\times P}$, where *D* represents the number of archetypes. The algorithm optimizes the parameters of *W* and *B* alternately, minimizing ${\Vert X{\prime} -WH\Vert }^{2}={\Vert X{\prime} -WBX{\prime} \Vert }^{2}$ subject to ${W}_{:,i} > 0\;\&\; {\sum }_{i=1}^{D}{W}_{:,i}=1$ and ${B}_{:,i} > 0\;\&\; {\sum }_{i=1}^{S}{B}_{:,i}=1$, where *S* spot counts and *D* archetypes are convex combinations of each other^74^. We applied Fisher separability analysis^80^ to infer the intrinsic dimension as its lower bound and iterated through different *K* values until the explained variance converges. We also implemented a hierarchical structure to fine tune the archetypes’ granularity with a resolution parameter *r* (ref. ^81^) (default set to 100). For archetype *a*_*i*_, *i* ∈ 2,…, *D*, if it resides within a Euclidean distance of *r* from any archetype *a*_*j*_, *j* ∈ 1,…, *i* − 1, we merge *a*_*i*_ with the closest *a*_*j*_. The archetypes distant from each other are kept after the shrinkage iteration and used in subsequent steps.

We define archetypal communities as the *r*-nearest neighbors (same as the resolution parameter) to each archetype by constructing *D* clusters. Next, for each cluster *i*, we identify the top 30 marker genes by performing a Wilcoxon rank-sum test between in-group and out-of-group spots with Scanpy^82^. We then refine cell state markers by assigning archetypal communities to the closest cell states. First, we align *D* archetypal communities with the best one-to-one matched *K* cell states with stable marriage matching^83^ and then append the archetypal marker genes to the given cell state. Next, we update the anchor spots according to the updated gene list. Alternatively, to find new cell states, we rank the archetypal clusters from the most distant to the least distant to the anchor spots of known cell states, and the archetypal clusters distant from all anchor spots represent potential new states for further study.

The overall archetypal analysis algorithm in Starfysh is summarized as follows:

1. Estimate the intrinsic dimension of the count matrix, and find *k* archetypes that identify the hypothesized purest spots.
2. Find the *N*-nearest neighbors of each archetype, and construct archetypal communities.
3. Find the most highly and differentially expressed genes for each archetypal community, and select the top *n* genes (default, *n* = 30) as the ‘archetypal marker genes’.
4. If the signature gene sets are provided, align the archetypal communities to the best matched known cell types, update the signature genes by appending archetypal marker genes to the aligned cell type and recalculate the anchors.
5. If the signature gene sets are absent, apply the archetypes and their corresponding marker genes as the signatures.

We found that archetypes alone are sufficient for disentangling major cell types but not fine-grained cell states (Supplementary Fig. 3e); however, when used as empirical priors to the deep generative model, they can guide the successful deconvolution of cell states (Supplementary Fig. 3a).

#### Starfysh structured variational inference

Starfysh uses variational inference to approximate the posterior. We first describe the inference procedure without integrating the histology variable *y*_*i*_. The posterior on variables *u*_*k*_ (cell states representations) are approximated by mean-field distributions *q*(*u*_*k*_), while the posterior on the variables *c*_*i*_ and *l*_*i*_ (cell state proportions and library size) are approximated by amortized mean-field distributions *q*(*c*_*i*_|*x*_*i*_) and *q*(*l*_*i*_|*x*_*i*_). Next, for each spot *i*, we use a specially structured variational distribution *q*(*z*_*i*_|*c*_*i*_, *x*_*i*_) that uses cell state proportions to sample the latent variables *z*_*i*_. Because each spot contains multiple cell states with proportions *c*_*i*_, the structured variational distribution is assumed to decompose as a combination of cell state-specific terms (denoted by *ζ*(*k*, *x*_*i*_) for each cell state *k*), weighted by the proportion of cell states *c*_*i*_. The variational family factorizes in the form $q(u,{c},{z},{l|x})={\prod }_{k=1}^{K}q({u}_{k}){\prod }_{i=1}^{n}q({c}_{i}|{x}_{i})q({l}_{i}|{x}_{i})q({z}_{i}|{c}_{i},{x}_{i}\;)$, parametrized by new variational parameters *m*_*k*_ and *v*_*k*_ and neural networks *λ*, *γ* and *ζ* as follows:

$$\begin{array}{ll}{\qquad\quad\,}q({u}_{k}) \, = \, {\rm{Normal}}({m}_{k},{v}_{k})\\ {\qquad\,\,}q({l}_{i}{\rm{|}}{x}_{i}) \, = \, {\rm{Normal}}\Big({\lambda }_{\mu }({x}_{i}),{\lambda }_{\sigma }({x}_{i})\Big)\\{\quad\,\,}q({c}_{i}{\rm{|}}{x}_{i}{\rm{;}}\,\alpha ) \, = \, {\rm{Dirichlet}}\Big(\alpha \cdot \gamma ({x}_{i})\Big)\\{\quad}q({z}_{i}{\rm{|}}{c}_{i},{x}_{i}) \, = \, {\rm{Normal}}\Big({\sum }_{k}{c}_{{ik}}\cdot {\zeta }_{\mu }(k,{x}_{i}),{\sum }_{k}{c}_{{ik}}\cdot {\zeta }_{\sigma }(k,{x}_{i})\Big).\end{array}$$

In summary, for each cell state *k*, the function *ζ*(*k*, *x*_*i*_) deconvolves the contribution of cell state *k* to the latent representation of *z*_*i*_. Each *z*_*i*_ is a combination of the cell state contributions *ζ*(*k*, *x*_*i*_) weighted by the proportions *c*_*i*_. The cell state proportions are inferred with the neural network *γ*, which is guided toward the prior to match the cell type gene sets. The prior strength parameter *α* also premultiplies the neural network *γ* to obtain a posterior of similar strength, which helps for the gradient optimization.

Next, the standard variational inference that maximizes the evidence lower bound (ELBO) is performed^84^. The ELBO in our case can be written as:

$$\begin{array}{ll}{\rm{ELBO}}\left(q\right) \, = \, \mathbb{E}_{q(z,c,l,u{\rm{|}}x)}\left[\log \frac{p\left(x,z,l,c,u{\rm{;}}\alpha ,A,\widetilde{l},\sigma \right)}{q(z,c,l,u{\rm{|}}x)}\right]\\ \qquad\qquad\;\,\, = \, \,\mathbb{E}_{q\left(z,c,l,u,{|x}\right)}[\log p(x{\rm{|}}z,l\;)]\\ \qquad\qquad\qquad\, \, -\mathbb{E}_{q\left(c,|,x\right)q\left(u\right)}\left[{D}_{{\rm{KL}}}\Big(q({z|c},x)\| p(z{\rm{|}}u,c{\rm{;}}\sigma )\Big)\right]\\ \qquad\qquad\qquad\, \, -{D}_{{\rm{KL}}}\Big(q({c|x}{\rm{;}}\alpha ){\rm{||}}p(c{\rm{;}}\alpha ,A)\Big)\\ \qquad\qquad\qquad\, \, -{D}_{{\rm{KL}}}\Big(q(l{\rm{|}}x){\rm{||}}p(l{\rm{;}}\widetilde{l})\Big)-{D}_{{\rm{KL}}}\Big(q(u){\rm{||}}p(u)\Big),\end{array}$$

where *D*_KL_(*p* || *q*) is the Kullback–Leibler divergence between distribution *p* and *q*, defined as *D*_KL_(*p* || *q*) = 𝔼_*p*(*x*)_[log *p*(*x*)/*q*(*x*)]. We find the *q* that maximizes the ELBO by running stochastic gradient descent.

#### Starfysh structured variational inference with histology integration

To integrate the histology in the inference method, we model the approximate posterior over the latent low-dimensional representation *z* with the PoE distributions (Supplementary Fig. 1a). For each spot *i*, we denote the view-specific encoders *qθ*_1_ (*z*_*i*_|*c*_*i*_, *x*_*i*_) and *qθ*_2_ (*z*_*i*_|*y*_*i*_) from the corresponding expression *x*_*i*_ and image patch *y*_*i*_, respectively. The expression view ${q}_{{\theta }_{1}}({z}_{i}|{c}_{i},{x}_{i})={\rm{Normal}}({\mu }_{1},{{\sigma }_{1}}^{2})$ is the same as described. For the histology view, *z*_*i*_ is approximated by amortized mean-field distribution ${q}_{{\theta }_{2}}({z}_{i}|\;{y}_{i})={\rm{Normal}}({\mu }_{2},{{\sigma }_{2}}^{2})={\rm{Normal}}({\xi }_{\mu }({y}_{i}),{\xi }_{\sigma }({y}_{i}))$ with a single-layer neural network $\xi$. For the joint latent variables ${z}_{i}$, the posterior distribution *q*(*z*_*i*_|*c*_*i*_, *x*_*i*_, *y*_*i*_) is parameterized as a product of view-specific Gaussian distributions as described in the original method^26^:

$${q}_{\theta }({z}_{i}{\rm{|}}{c}_{i},{x}_{i},{y}_{i})=\frac{{\mu }_{1}/{{\sigma }_{1}}^{2}+{\mu }_{2}/{{\sigma }_{2}}^{2}}{1/{{\sigma }_{1}}^{2}+1/{{\sigma }_{2}}^{2}}.$$

The previous ELBO can be updated with this new variational approximation for the joint modeling of histology and transcriptome. We leverage the information bottleneck approach^26^ to optimize the joint ELBO as well as the view-specific marginal ELBOs through a single objective function ${{\mathscr{L}}}_{{\rm{total}}}={{\mathscr{L}}}_{{\rm{joint}}}+a\cdot {{\mathscr{L}}}_{{\rm{marginal}}}$, where:

$$\begin{array}{ll}\quad{{\mathscr{L}}}_{{\rm{joint}}} \, = \, {\rm{ELBO}}({q}_{\theta })={E}_{{q}_{\theta }(z,l,c,u{\rm{|}}x,y)}\log \frac{p(x,y,z,l,c,u{\rm{;}}\sigma )}{{q}_{\theta }(z,l,c,u{\rm{|}}x,y)}\\\qquad\qquad= \, {E}_{{q}_{\theta }(z{\rm{|}}x,y){q}_{\theta }(l{\rm{|}}x)}\,\log p(x{\rm{|}}z,l)+{E}_{{q}_{\theta }(z{\rm{|}}x,y)}\log p(y{\rm{|}}z)\\ \qquad\quad\qquad-\,{E}_{{q}_{\theta }(c{\rm{|}}x){q}_{\theta }(u)}{D}_{{\rm{KL}}}\Big({q}_{\theta }(z{\rm{|}}c,x,y)\| p(z{\rm{|}}c,u{\rm{;}}\sigma )\Big)\\ {{\mathscr{L}}}_{{\rm{marginal}}} \, = \, {\rm{ELBO}}({q}_{{\theta }_{1}})+{\rm{ELBO}}({q}_{{\theta }_{2}}).\end{array}$$

The variational family for the joint objective function is factorized as ${q}_{\theta }(z,{l},{c},{u|x},{y})={q}_{\theta }({z|x},{y}){q}_{\theta }({l}|\;{y}){q}_{\theta }({c|x}){q}_{\theta }(u)$. Hyperparameter *a* (set by default as 5) balances the weights between joint and view-specific objectives^26^. The expression view ${\rm{ELBO}}({q}_{{\theta }_{1}})$ remains the same with above, and the histology view ${\rm{ELBO}}({q}_{{\theta }_{2}})$ is written as:

$$\begin{array}{ll}{\rm{ELBO}}({q}_{{\theta }_{2}}) \, = \, {E}_{{q}_{{\theta }_{2}}(z{\rm{|}}y)}\log \frac{p(y,z,c,u{\rm{;}}\sigma )}{{q}_{{\theta }_{2}}(z{\rm{|}}y)}\\\qquad\qquad\quad\,= \, {E}_{{q}_{{\theta }_{2}}(z{\rm{|}}y)}\log p(y{\rm{|}}z)-{E}_{{q}_{{\theta }_{2}}\left(c{|}y\right){q}_{{\theta }_{2}}\left(u\right)}{D}_{{\rm{KL}}}\left({q}_{{\theta }_{2}}(z{\rm{|}}\;y){\rm{||}}p(z{\rm{|}}u,c{\rm{;}}\,\sigma )\right).\end{array}$$

The same conditional prior *p*(*z*|*c*, *u*; *σ*) is applied across the joint and view-specific ELBOs. We find the $\{{q}_{\theta },{q}_{{\theta }_{1}},{q}_{{\theta }_{2}}\}$ that maximize ${{\mathscr{L}}}_{{\rm{total}}}$ by running stochastic gradient descent.

#### Starfysh implementation

The Starfysh model is implemented as a Python package using PyTorch^85^ with the Adam^86^ optimizer. The model by default is trained for 200 epochs with a learning rate at 0.001. During the training, the learning rate decays, guided by an exponential scheduler with the multiplicative factor set as 0.98. Kaiming initialization is applied to all neural network parameters. Hyperparameters are adjustable in the package.

#### Prediction of cell state-specific expression

To predict cell state-specific expression, we use the decoder in which the parameters have been learned and optimized by the variational inference. The proportion *c*_*i*_ is adjusted to 1 for a specific cell state and 0 for other cell states. Reconstructed expression and histology are considered as cell state-specific expression and histology.

#### Integration of multiple samples

To effectively integrate multiple samples, Starfysh initially identifies anchors in each sample by combining spots enriched for cell types and archetypal communities. The gene markers for each sample are then updated based on the newly defined anchors. Subsequently, we aggregate the gene markers for each cell type across all samples. These updated markers are used to calculate priors for the cell state proportions when fitting to all samples simultaneously. Priors for library size are separately calculated for spots in each sample. Finally, transcriptomic counts along with their corresponding histological patches are incorporated as inputs to train an integrated model, synergizing data across samples.

### Simulation of ST data

We construct our ST simulations using mixtures of scRNA-seq data previously collected from primary TNBC tumor tissues (CID44971_TNBC)^18^ with different levels of cell type granularities.

#### Spatially dependent simulation

To address spatial dependencies among neighboring spots, we adopt the pipeline from Cell2location^8^. Specifically, synthetic ST spots are defined on a 50 × 50-pixel grid. For the major cell type simulation, we select five cell types (CAFs, cancer epithelial cells, myeloid cells, normal epithelial cells, T cells) from the reference scRNA-seq data and simulate their spatial proportions with separate 2D Gaussian process models (Supplementary Fig. 2a). We further assign an expected library size for each spot with a γ distribution fitted from the real ST dataset, representing the spatial variation of capture rates among spots. For each spot, we then sample single-cell transcriptomes from the reference by searching for candidate cells with a library size closest to the expected library size. We follow the same procedure to generate another ten-cell type simulation with finer cell states: basal cells, inflammatory CAFs, myofibroblast CAFs, endothelial cells, immature PVL cells, central memory T cells, T_reg_ cells, activated CD8^+^ T cells, memory B cells and plasmacytoid dendritic cells.

#### Simulation with paired histology images

We further generate pseudo-histology images paired with the aforementioned major cell type simulation to verify multimodel integration. Specifically, we design a supervised encoder–decoder neural network model (Supplementary Fig. 1c), with real ST expression as input and their histology images as output. First, the expression matrix is projected to a low-dimensional latent space with a ResNet18 encoder, and the histology image is reconstructed with a standard linear decoder with dimension transformation. Two thousand image patches and corresponding expression matrices were trained from 14 ST samples, and an extra 500 images patches were used for held-out validation. The learning rate was set as 0.001 with the Adam optimizer for training. Mean-squared loss was used to fit the predictions to the real ST images. The final paired synthetic histology images were generated by running the trained model.

#### Signature gene set retrieval in simulated data

For fair benchmarking not favoring Starfysh, we build the signature gene sets in an unbiased fashion by choosing the top 30 differentially expressed genes for each cell type (highest log (FC) scores) across 20 breast cancer scRNA-seq samples reported by Wu et al.^18^.

### Benchmarking of Starfysh and comparison to other methods with simulated ST data

We benchmarked Starfysh against reference-based (DestVI, Cell2location, Tangram, BayesPrism) and reference-free (CARD, BayesTME, STdeconvolve) deconvolution methods with the aforementioned simulations. For the reference-based method, we used paired scRNA-seq data for sample TNBC sample CID44971 as the reference. For reference-free methods without inferred cell state annotations, we report the best alignment with the ground truth proportions upon permutation.

For each deconvolution, we trained Starfysh with three independent restarts and selected the model with the lowest ${{\mathscr{L}}}_{c}$. The variational mean *q*(*c*_*ik*_*|x*_*i*_; *α*) is used as the inferred cell state proportions.

For BayesPrism, we followed the tutorial on the BayesPrism website: https://www.bayesprism.org/pages/tutorial_deconvolution. We subsetted the common protein-coding genes between the scRNA-seq and ST data with highly variable gene selection by default. We ran the BayesPrism Gibbs sampler ‘run.prism’ with four cores and extracted the updated cell type fractions *θ*_*n*_ for deconvolution.

For Cell2location, we followed the tutorial on the Cell2location website: https://cell2location.readthedocs.io/en/latest/notebooks/cell2location_tutorial.html. We trained the reference regression with 1,000 epochs and spatial mapping models with 10,000 epochs, in which ELBO losses were ensured. The normalized 5% quantile values of the posterior distribution ${\hat{w}}_{{sf}}=\frac{{w}_{{sf}}}{{\varSigma }_{f}{w}_{{sf}}}$ were used for deconvolution.

For DestVI, we followed the DestVI tutorial with default parameters at https://docs.scvi-tools.org/en/stable/tutorials/notebooks/DestVI_tutorial.html.

For Tangram, we followed the Tangram tutorial using default settings: https://github.com/broadinstitute/Tangram/blob/master/tutorial_tangram_with_squidpy.ipynb. We found the optimal alignment for scRNA-seq profiles with 1,000 epochs.

For CARD (reference free), we followed the CARD reference-free tutorial: https://yingma0107.github.io/CARD/documentation/04_CARD_Example.html. Default settings were used to generate cell type proportions (minCountGene = 100 and minCountSpot = 5).

BayesTME (reference free) deconvolves cell types with a hierarchical probabilistic model that corrects technical artifacts. We followed the official BayesTME tutorial with default parameters: https://github.com/tansey-lab/bayestme/blob/main/notebooks/deconvolution.ipynb.

For STdeconvolve (reference free), we followed the tutorial on the STdeconvolve website (https://jef.works/STdeconvolve/) and selected the top 1,000 overdispersed genes from the input matrix. We set the optimal number of cell types *K* to 5 and 10 for the major and fine cell type simulations, respectively. The predicted cell type proportions were obtained from the output ‘deconProp’.

#### Quantification of performance in deconvolution of cell types

The performance of each method was summarized by the RMSE and Jensen–Shannon divergence (JSD) against the ground truth to quantify per-spot accuracy (Supplementary Fig. 2d,e):

$$\begin{array}{ll}{\rm{RMSE}}\left({{c}_{i}}^{{gt}},{{c}_{i}}^{{\rm{pred}}}\right) \, = \, \sqrt{\frac{\mathop{\sum }\nolimits_{k=1}^{K}{\left({{c}_{{ik}}}^{{gt}}-{{c}_{{ik}}}^{{\rm{pred}}}\right)}^{2}}{K}}\\\quad\; {\rm{JSD}}\left({{c}_{i}}^{{gt}},{{c}_{i}}^{{\rm{pred}}}\right) \, = \, \frac{1}{2}{D}_{{\rm{KL}}}\left({{c}_{i}}^{{gt}}{\rm{||}}{{c}_{i}}^{{\rm{pred}}}\right)+\frac{1}{2}{D}_{{\rm{KL}}}\left({{c}_{i}}^{{\rm{pred}}}{\rm{||}}{{c}_{i}}^{{gt}}\right),\end{array}$$

where ${{c}_{i}}^{{gt}},{{c}_{i}}^{{\rm{pred}}}\in {\varDelta }^{K}$ represent the ground truth and predicted cell type compositions in spot *i*. We report the average RMSE across all spots as the overall performance for each method (Fig. 1d).

### Benchmarking of Starfysh and comparison to other methods with real ST data

We further benchmarked Starfysh with reference-based (Cell2loation and BayesPrism) and reference-free (STdeconvolve) deconvolution methods on TNBC sample CID44971 ST data (Supplementary Fig. 3b–d). We calculated the correlation $A\in {{\mathbb{R}}}^{K\times K}$ between the average expression of gene sets (normalized to sum to 1 per spot) (Supplementary Table 2) and the deconvolution profile for each cell state:

$$\begin{array}{ccc}{A}_{{kl}} & = & {\rm{Corr}}\Big({{c}_{:k}}^{{\rm{sig}}},{{c}_{:l}}^{{\rm{pred}}}\Big)\\ {\bar{c}}_{{ik}} & = & \frac{{\sum }_{g}{x}_{{ig}}\cdot {s}_{{kg}}}{{\sum }_{g}{s}_{{kg}}},{c}_{{ik}}^{{\rm{sig}}}=\frac{{\bar{c}}_{{ik}}}{\mathop{\sum }\nolimits_{k=1}^{K}{\bar{c}}_{{ik}}},\end{array}$$

where ${c}_{:k}^{{\rm{sig}}},{c}_{:l}^{{\rm{pred}}}\in {{\mathbb{R}}}^{S}$ represent signature marker’s expression and deconvolution proportions for cell states *k* and *l*, respectively.

For Starfysh, we followed the same procedure from the simulation benchmark and reported the variational mean *q*(*c*_*ik*_|*x*_*i*_; *α*) as the deconvolution profile.

For both BayesPrism and Cell2location, we followed the same procedures as the simulation benchmark, except for replacing the synthetic ST data with real ST data from TNBC sample CID44971. We applied the TNBC sample CID44971 scRNA-seq annotation from the ‘subset’ classification tier from Wu et al.^18^. For correlation calculation, intersections between single-cell annotations^18^ and our signature cell types are shown, as BayesPrism and Cell2location only deconvolve cell types that appear in the reference.

For STdeconvolve, we iterated the number of factors (*k*) from 20 to 30 and chose the optimal *k* as 30 given the lowest perplexity following the official tutorial. Because STdeconvolve does not explicitly annotate factors, we performed hierarchical clustering between factors (*x* axis) and cell types (*y* axis).

We applied archetypal analysis (Starfysh) to the ST data and identified 18 distinct archetypes. We reported the overlapping percentage between anchor spots and archetypal communities for each cell state (Supplementary Fig. 3e).

#### Quantification of performance in deconvolution of cell states in real ST data

Performance in disentangling cell states was evaluated using the Frobenius norm $d={\Vert A-{A}^{\rm{sig}}\Vert }_{F}$ as the distance between the deconvolution-to-signature correlation *A* to the ‘reference’ matrix ${{A}_{{kl}}}^{{\rm{sig}}}={\rm{Corr}}({{c}_{:k}}^{k},{{c}_{:l}}^{l})$, defined as the correlation between signature expressions across cell states. To ensure a fair comparison across reference-based and reference-free methods, we reported a Frobenius norm distance computed as follows: for each method, (1) 1,000 10 × 10 submatrices {*A*^(1)^,…, *A*^(1,000)^} were sampled from the original correlation matrix *A* without replacement with randomly permuted cell states; (2) an array of Frobenius norm distance $\overrightarrow{d}=({d}^{(1)},\ldots ,{d}^{(1,000)}),\,{d}^{(i)}={\Vert {A}^{(i)}-{A}^{{\rm{sig}}(i)}\Vert }_{F}$ was computed; and (3) we reported the average value of ${d}_{i}$ in Supplementary Fig. 3a–d. To test the improvement of Starfysh, we performed a Mann–Whitney *U*-test between the distance array of Starfysh against the combination of all other methods (BayesPrism, Cell2location, STdeconvolve).

For reference-free methods in which the number of inferred factors and the number of cell types may differ, we permuted the correlation matrix such that each cell type (row) was aligned with the factor (column) with the highest correlation score, where the diagonal entries were sorted in descending fashion.

#### Runtime comparison across deconvolution methods on real ST data

Runtimes of the core deconvolution function in each method were measured on the same machine with 12-core AMD Ryzen 9 3900X CPU and a GeForce RTX 2080 GPU:

- Starfysh: run_starfysh (GPU-enabled)
- BayesPrism: run.prism
- Cell2location: RegressionModel.train(),Cell2location.train() (GPU-enabled)
- STdeconvolve: fitLDA

### Starfysh validation with Xenium-mapped ST data

We further applied Starfysh to a recent breast cancer ST dataset, for which integrated multicellular (Visium, replicate 1) and subcellular in situ (Xenium) spatial technologies were performed on the same formalin-fixed, paraffin-embedded tissue blocks^29^. We first aligned the Visium H&E images and spots to the paired Xenium H&E images with SIFT registration^87^. The ground truth deconvolution profile was then constructed by assigning spots to their corresponding Xenium cells annotated by Janesick et al.^29^. A total of 2,567 spots with nine major cell types were kept after filtering out spots with unannotated cells (Supplementary Fig. 4a). Benchmarking metrics were computed the same way as for the simulation data. Original datasets as well as the signatures used by Starfysh are publicly available at https://www.10xgenomics.com/support/in-situ-gene-expression/documentation/steps/onboard-analysis/at-a-glance-xenium-output-files.

### Starfysh validation with ST data of mouse cortex and human lymph node

We applied Starfysh to mouse brain data adapted from Cell2location^8^ and used the marker genes provided by the paper, which are collected from literature with known regional marker genes or the Allen Brain Atlas. Histology integration is applied in this dataset also. Starfysh successfully recognized enriched regions such as Bergmann glia of the cerebellum (ACBG), cortex pyramidal layer 6 (TEGLU3), the basolateral amygdala (TEGLU22) and the hippocampus (TEGLU24) (TEGLU, telencephalon projecting excitatory neurons; Supplementary Fig. 6a). Starfysh also reconstructed the histology data resembling original images (Supplementary Fig. 6b). Inferred spatial hubs recapitulated the brain regions identified from Cell2location (Supplementary Fig. 6c), such as the thalamus (hubs 8 and 9), the hypothalamus (hubs 7 and 19), the cortex (hubs 0, 1 and 5), the amygdala (hubs 6 and 12), the hippocampus (hubs 10 and 20), the striatum (hub 11) and white matter (hubs 4 and 13).

We also applied Starfysh to human lymph nodes with gene signatures from a comprehensive atlas of 34 cell types in human lymphoid organs^88–90^. The results recapitulated the identification of T cell and B cell zones and germinal centers with dark-zone, light-zone and follicular dendritic cells reported as in Cell2location (Supplementary Fig. 6d). Starfysh also distinguished blood vessel zones, similar to the results in Cell2location. The identified spatial hubs (Supplementary Fig. 6e) showed similar alignment with Cell2location (scRNA-seq reference based)-defined spatial clusters through the MIC (Supplementary Fig. 6e,f).

### Starfysh validation with spatiotemporal analysis of prostate cancer

To evaluate Starfysh’s power in unraveling mechanisms in more complicated scenarios, such as spatiotemporal ST datasets, we applied it to ST datasets from prostate cancer tissues undergoing AD therapy^30^. ST profiling provided a unique perspective on the tumor and microenvironment in this specific prostate cancer, called castration-resistant PCa, a type with challenging tumor grade classification and unpredictable treatment outcomes.

Unlike the published study that used spatial transcriptome decomposition^91^ for patient-by-patient spatiotemporal analysis, Starfysh demonstrated superior efficacy in identifying more interpretable niches. It integrated samples from three patients with four biopsies each and two biological replicates per biopsy and samples from both pretreatment and post-treatment stages (Supplementary Fig. 7a,b).

UMAP visualization of the joint space of inferred cell type proportion highlighted specific features such as clustering of tumor cells, immune cells and stromal cells (Supplementary Fig. 7c). We defined 17 hubs within this joint space (Supplementary Fig. 7d), and their spatial distribution illustrated changes before and after AD treatment across patients and revealed similarities across replicates (Supplementary Fig. 7e). Each hub represented aggregations of specific cell types (Supplementary Fig. 7f), with ranking based on tumor cell proportions including tumor-enriched hubs (Supplementary Fig. 7g). For instance, hub 0 was enriched with prostate cancer and stromal cells such as CAFs and perivascular cells, whereas hub 1 had predominantly cancer cells.

Patient-specific variances were evident in the composition of these hubs, particularly in their response to AD treatment. Starfysh’s analysis aligned with clinical data, categorizing patients into responders (patient 1), moderate responders (patient 2) and nonresponders (patient 3). For example, tumor-enriched hub 0 predominated in the nonresponder (patient 3), while hub 15 was specific to the moderate responder (patient 2) (Supplementary Fig. 7h). Differential gene expression analysis of hub 0 revealed enrichment in EMT pathways and myogenesis, indicating resistance to treatment (Supplementary Fig. 7h,i). Additionally, hub 0 exhibited low AR activity (Supplementary Fig. 7j), aligning with findings that stromal cells adjacent to resistant clusters lacked androgen receptor expression and were enriched with EMT pathways. Starfysh not only identified similar regions but also highlighted specific cell type infiltrations, including those of CAFs and perivascular cells. Moreover, ST data indicated a trend from tumor hubs (hubs 13 and 15) to hub 0 upon treatment, which is beneficial for interpatient analysis.

### Breast tumor ST data collection and analysis

#### Sample collection and preparation

Tissues were collected from women undergoing surgery for primary breast cancer. All samples were obtained after informed consent and approval from the institutional review board at Memorial Sloan Kettering Cancer Center. Samples were obtained using standard-of-care procedures. The samples were embedded fresh in Scigen Tissue-Plus O.C.T. Compound (Fisher Scientific) and stored at −80 °C before sectioning. Cryosections (10 μm) were mounted on Visium spatial gene expression slides (10x Genomics, 1000184). Two individual tumors were mounted in duplicate on the four 6.5-mm × 6.5-mm capture areas. The samples were processed as described in the manufacturer’s protocols.

#### Spatial transcriptomics by 10x Genomics Visium

Visium Spatial Gene Expression slides prepared by the Molecular Cytology Core at MSKCC were permeabilized at 37 °C for 6 min, and polyadenylated mRNA was captured by oligonucleotides bound to the slides. Reverse transcription, second-strand synthesis, complementary DNA (cDNA) amplification and library preparation proceeded using the Visium Spatial Gene Expression Slide & Reagent Kit (10x Genomics, 1000184) according to the manufacturer’s protocol. After evaluation by real-time PCR, cDNA amplification included 13–14 cycles; sequencing libraries were prepared with 15 cycles of PCR. Indexed libraries were pooled in an equimolar fashion and sequenced on a NovaSeq 6000 instrument in a PE28/120 run using the NovaSeq 6000 SP Reagent Kit (200 cycles) (Illumina). An average of 228 million paired reads were generated per sample.

Tissues were stained with H&E, and slides were scanned on a Pannoramic MIDI scanner (3DHISTECH) using a ×20, 0.8-NA objective.

Quality metrics for the collected ST data are shown in Supplementary Table 5.

#### CODEX data collection and preprocessing

Four fresh-frozen samples, adjacent slides with P3A_MBC, P3B_MBC, P4A_MBC and P4B_MBC, were processed for PhenoCycler (CODEX) imaging in Enable Lab (https://www.enablemedicine.com). Samples were prepared and stained, and images were acquired following CODEX User Manual Rev C (https://www.akoyabio.com) at Enable Medicine. Twenty-three antibodies were used for staining in this study (Supplementary Table 6). Image data were preprocessed using commercial software (Enable Medicine).

### Analysis of ST data from breast tumor tissues

#### Data preprocessing

Starfysh is compatible with Scanpy^82^ and preprocesses the raw count matrix as input without normalization after filtering out ribosomal and mitochondrial genes. To account for expression sparsity and noise, we selected the top 2,000 highly variable genes including specified marker genes.

#### Identification of tumor-associated anchors

Tumor-associated archetypes were defined as the anchor spots highly associated with tumor cell types. First, an initial set of cell state-enriched spots (for example, 60 spots for each cell state) and *M* archetypes were identified based on the provided marker gene list and the PCHA algorithm, respectively. Because archetypes are vertices non-overlapping with observed data, the *r* = 20 nearest-neighbor spots for each archetype were identified, obtaining a set of ‘archetypal communities’ as a 20 × *M* matrix. Next, we aligned archetypal communities with the best one-to-one matched *K* cell states with the stable marriage algorithm. Anchor spots were then updated based on the new marker gene list. The final anchors that are associated with any tumor cell gene set (including TNBC, MBC, LumA, LumB and ER^+^) were considered as TAAs (Figs. 2d,h and 4c).

#### Diffusion component analysis

Diffusion components were computed using normalized gene counts as the input. Computation was performed with the Scanpy package. Scanpy computes diffusion components by first constructing a nearest-neighbor graph from the high-dimensional input data. Next, it simulates a diffusion process on the graph.

#### Definition of hubs

Hubs were defined as groups of spots with a similar composition of cell states. To integrate ST samples from different patients, anchors were defined on merged data from all samples, and Starfysh then inferred the cell state proportion and latent variables for each spot in each sample using the same anchor set. Spots were then clustered according to the inferred cell state proportion using PhenoGraph clustering (Supplementary Fig. 11c).

#### Entropy of spots

We used an entropy-based metric previously used for batch correction in single-cell data^35^ for evaluating the integration of samples. The Shannon entropy of spots denotes mixing of spots across samples. Specifically, we constructed a kNN graph for each spot *i* to determine its nearest neighbors using Euclidean distance in the Starfysh latent space (*z*). These nearest-neighbor spots formed a distribution of patients $(m\in \{1,\ldots 14\}\,)$ for the overall 14 patients studied in this paper, represented as ${{e}_{{i}^{}}}^{m}$. The Shannon entropy is calculated as ${H}_{i}=-{\sum }_{m=1}^{14}{{e}_{i}}^{m}\log {{e}_{i}}^{m}$. Higher entropy represents higher localized sample mixing across patients (Fig. 3d).

#### Kendall’s *τ* correlation

Kendall’s *τ* correlation is a metric for measuring the ordinal association between two measured quantities. We used this metric to quantify the heterogeneity of TAAs. Genes for TAAs were ranked based on differential expression scores for each sample. Samples having similar TAAs were assumed to have a similar rank of differential genes, thus having higher scores of Kendall’s *τ* correlation (Fig. 2p).

#### Copy number variation

Copy number variation was performed following the instructions for inferCNV (https://github.com/broadinstitute/inferCNV). The inferred copy number variation cluster lineage was plotted as a dendrogram tree using toytree^92^.

#### Definition of intratumoral, peritumoral and stromal regions

We applied Starfysh to TNBC and MBC samples to avoid the bias introduced by those ER^+^ samples and redefined the hubs among six TNBC and four MBC samples. Intratumoral regions were defined as hubs with the mean of inferred proportions of all tumor states being larger than 0.2 (Supplementary Fig. 13b). Histology information was also considered to confirm the enrichment of tumor cells in these regions. Other hubs were ranked by the average distance (unit, pixel) to intratumoral hubs. With the incorporation of histology and total proportion of immune cells and stromal cells, hub 8 was considered as the boundary between peritumoral regions and stromal regions (Supplementary Fig. 13c). To summarize, hubs 5, 2, 11 and 12 were considered as intratumoral hubs, hubs 0, 9, 3, 6 and 8 were considered as peritumoral hubs, and hubs 1, 7, 4 and 10 were recognized as stromal hubs. Notably, the determined peritumoral regions were shared across all samples, while some intratumoral regions and stromal regions were sample specific (Supplementary Fig. 13a,d and Fig. 4b).

#### Spatial correlation

To measure colocalization between cell states, we slightly modified the spatial cross-correlation index (SCI)^54^. SCI is defined as:

$${\rm{SCI}}\Big({S}_{x},{S}_{y}\Big)=\frac{N}{2\mathop{\sum }\nolimits_{i}^{N}\mathop{\sum }\nolimits_{j}^{N}{\tau }_{{ij}}}\frac{\mathop{\sum }\nolimits_{i}^{N}\mathop{\sum }\nolimits_{j}^{N}{\tau }_{{ij}}({x}_{i}-\bar{x})(\;{y}_{i}-\bar{y})}{\sqrt{\mathop{\sum }\nolimits_{i}^{N}{({x}_{i}-\bar{x})}^{2}}\sqrt{\mathop{\sum }\nolimits_{j}^{N}{(\;{y}_{j}-\bar{y})}^{2}}},$$

where *x* and *y* denote the predicted proportion for two cell states *S*_*x*_ and *S*_*y*_, *i* and $j\in [1,\mathrm{.}.,N]$ are indexes of spots within a certain hub and $\bar{x},\bar{y}$ are the mean proportion of two cell states in the hubs. We defined the weight matrix $\tau$ as information between adjacent neighbors, as *τ*_*ij*_ = 1 if the coordinate distance of spot *i* and spot *j* was less than $\sqrt{3}$, else *w*_*ij*_ = 0.

#### Inference of intercellular ligand–receptor interactions

To investigate the intercellular interactions in a hub, the top 5% spots with the highest inferred proportion of each cell state in the hub were selected. CellPhoneDB^55^ was then applied to the selected spots with normalized gene expression. Visualization was performed with the Sankey diagram with plotly and the Circos plot^93^.

#### Diffusion map analysis with intratumoral hubs

Intratumoral hubs were selected for diffusion map analysis (Fig. 2h), and diffusion map components showing gradients between intratumoral hubs were chosen. Diffusion map coordinates were used as inputs for the trajectory inference algorithm SCORPIUS^49^. Modules of genes that significantly (*q* values < 0.05) contributed to the trajectory of transitions between tumor hubs were identified (Fig. 2i). Over-representation analysis was conducted to understand the biological processes via the Python package gseapy with gene sets including KEGG_2021_Human, GO_Biological_Process_2021 and Hallmark.

#### Genes with diffused expression patterns

T_reg_-enriched (proportion > 0.05) spots in intratumoral hubs were selected, and the distance between all spots to the selected spots was calculated with the ‘sklearn.neighbors’ Python package with the function KDTree. For each gene, expression of spots with the same distance was averaged and smoothed with a window size of 7 for each sample. The mean and s.d. of expression across all samples were computed and smoothed with ‘Gaussian_filter1d(sigma = 1.5)’ with the Python package SciPy (mean and s.d. are shown as a solid line and shaded area in Fig. 4i).

#### CODEX data analysis

Raw CODEX images were segmented to enable cell-level quantification from biomarker signals. The results were then checked with quality control to filter out segmentation artifacts. The data thus were transformed as *a U* × *P* matrix, where *U* is the number of single cells detected in the CODEX images and *P* represents the number of antibodies profiled. The data were then processed by quantile normalization, asinh transform and *z*-score normalization. PCA, neighbor graphs and UMAP were performed sequentially on single-cell CODEX data (Supplementary Fig. 15a). Annotations of cell types were based on the clustering and distribution of normalized CODEX data such as Ki67 and CD3 expression (Supplementary Fig. 15b,c and Supplementary Table 6). Annotations were validated with a dendrogram tree of the clusters (Supplementary Fig. 15d). The single-cell CODEX was also visualized in the spatial arrangement aligning with the histology and ST Visium data (Supplementary Fig. 15e and Fig. 5c).

### Spatial profiling of T cell receptors

To capture spatial TCR clonotype information, we adapted an established protocol that allows spatial mapping of TCRs from cDNA libraries of our samples^46^. The process involves three qPCR steps: (1) the first step begins with 43 pooled TCRB primers and the truncated read 1 primer (2 µl cDNA, 1 µl of each forward and reverse primers and 12.5 µl NEBNext Master Mix, 0.5 µl SYBR and 8 µl water). (2) The second step uses 43 TCRB primers with R2 sequences and the truncated read 1 primer with 1 µl of the PCR product from step 1. (3) The third step involves indexed TruSeq P5 primers and indexed Nextera P7 primers, with 1 µl of the PCR product from step 2. All PCR steps were stopped before the plateau phase, and the PCR products were cleaned with 0.8× AMPure beads and eluted in 50 µl.

Sequencing was conducted on an Illumina NextSeq 500 instrument with the following cycle settings: R1 28, I1 10, I2 10, R2 110. Clonotype analyses were performed with MiXCR.

The PCR cycling conditions are as follows: initial denaturation, 98 °C for 3 min; denaturation, 98 °C for 15 s; annealing, 62 °C (72 °C for qPCR step 3) for 20 s; extension, 72 °C for 1 min; repeat of the denaturation step to the extension step before the plateaus phase; final extension, 72 °C for 1 min.

We further provide the full spatial TCR primer sequences in Supplementary Table 8.

### Reporting summary

Further information on research design is available in the Nature Portfolio Reporting Summary linked to this article.

## Online content

Any methods, additional references, Nature Portfolio reporting summaries, source data, extended data, supplementary information, acknowledgements, peer review information; details of author contributions and competing interests; and statements of data and code availability are available at 10.1038/s41587-024-02173-8.

## Supplementary information

Supplementary Information Supplementary Table 1 and Figs. 1–15.

Reporting Summary

Supplementary Table 2 Markers and gene sets. Signature gene sets for Starfysh input with annotated cell types and cell states in patients with breast cancer.

Supplementary Table 3 Cell state proportions inferred by Starfysh.

Supplementary Table 4 Metabolic signatures. Gene sets used to investigate metabolic signatures.

Supplementary Table 5 ST quality-control metrics. Important quality-control parameters for ST.

Supplementary Table 6 Antibody panels profiled with CODEX in MBC samples.

Supplementary Table 7 Markers and gene sets for mouse cortex and human lymph nodes.

Supplementary Table 8 Primer sequences for the spatial TCR experiment.

## Data Availability

The raw data generated for this study can be accessed in the Gene Expression Omnibus under accession number GSE218951. CODEX data are available in figshare (10.6084/m9.figshare.25137320) (ref. ^94^). The public breast cancer dataset from Wu et al. was downloaded from accession number GSE176078. Public mouse brain and lymph node datasets from Kleshchevnikov et al. are available in ArrayExpress under accession number E-MTAB-11114. Public prostate cancer data are available in Mendeley Data (10.17632/mdt8n2xgf4.1) (ref. ^95^).

## Code availability

The Starfysh package and code to reproduce the results in this study are available in the GitHub repositories at https://github.com/azizilab/starfysh (ref. ^96^) and https://github.com/azizilab/starfysh_reproducibility (ref. ^97^) and deposited at Zenodo (10.5281/zenodo.10460548) (ref. ^98^). The reference implementation of DestVI, RCTD and BayesTME, along with the accompanying tutorials, is also available at the GitHub repository.
