# Supplementary material for: Starfysh integrates spatial transcriptomic and histologic data to reveal heterogeneous tumor–immune hubs
Source: Nat Biotechnol. 2024 Mar 21;43(2):223–35. doi: 10.1038/s41587-024-02173-8 (PMC11415552; doi:10.1038/s41587-024-02173-8)
Supplement: Supplementary file 1 — Supplementary Table 1 and Figs. 1–15. [file 41587_2024_2173_MOESM1_ESM.pdf]

nature biotechnology

Article

https://doi.org/10.1038/s41587-024-02173-8

Starfysh integrates spatial transcriptomic and histologic data to reveal heterogeneous tumor–immune hubs

In the format provided by the authors and unedited

Supplementary table 1. Patient clinical information

Patient ID	Replicates	ER	PR	Her2	Age	Subtype
P1_ER	2	95	80	-	70	Ductal (invasive)
P2_TNBC	2	2	0	-	84	Ductal (invasive)
P3_MBC	2	0	0	-	71	Metaplastic (maxing producing)
P4_MBC	2	0	0	-	52	Metaplastic (matric producing)

Supplementary table 2. Marker genesets for tumor epithelial, immune, and stromal cells in breast tumor tissues

Supplementary table 3. Inferred cell type proportions by Starfysh

Supplementary table 4. Gene sets for metabolic pathways

Supplementary table 5. Spatial transcriptomics quality control metrics

Supplementary table 6. Antibody panels profiled with CODEX in MBC samples

Supplementary table 7. Marker genesets for mouse cortex and human lymph nodes

Supplementary table 8. Primer sequences for Spatial TCR experiment

Supplementary tables 2-8 are included in the supplementary material.

Supplementary figures

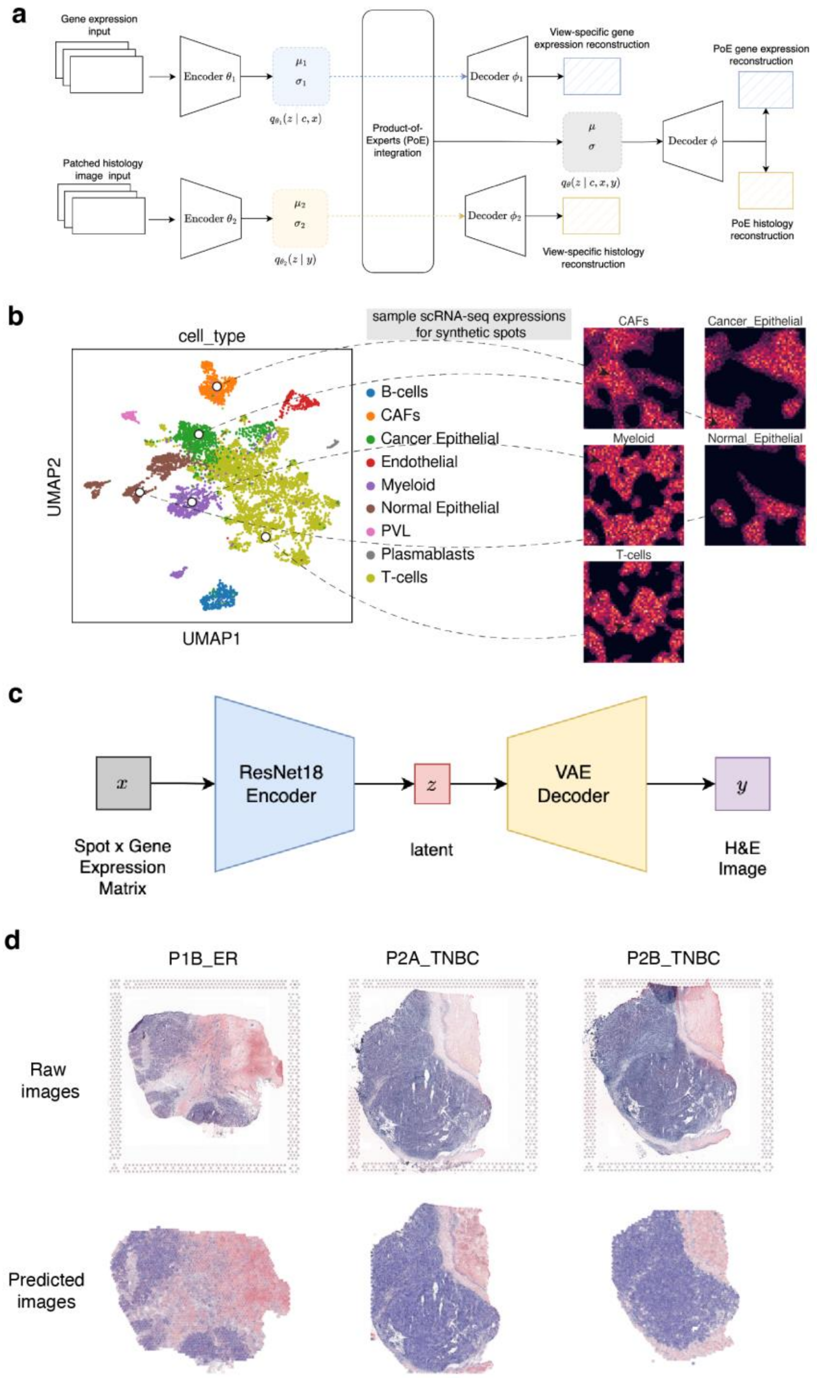

Supplementary Figure 1. Schematic representation of Starfysh histology integration and spatial image simulations. (a) Architecture of Product-of-Expert (PoE) integration with histology image and transcriptomic data. **(b)** Simulating ST data from primary breast tumor tissues. We sampled cell types from real single-cell matched data (TNBC sample CID44971) to construct gene expressions and proportions of the synthetic spots with spatial dependencies using a 2D Gaussian Process (Methods). **(c)-(d)** Simulating histology imaging data from gene expression. **(c)** The model architecture to simulate histology images from gene expression. **(d)** Example results on predicted images from gene expression data compared to ground truth histology images.

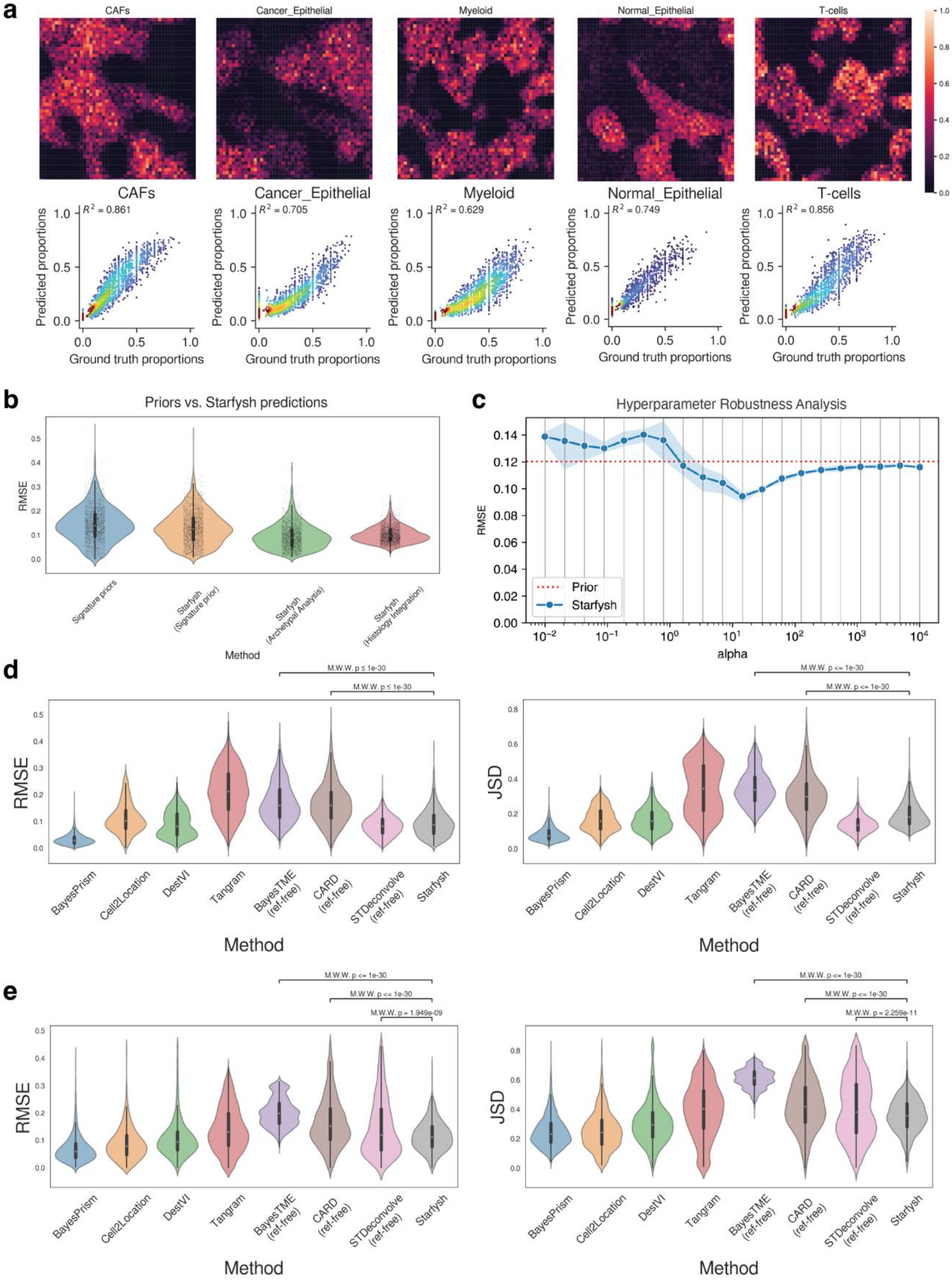

a
CAFs
Cancer_Epithelial
Myeloid
Normal_Epithelial
T-cells
$R^2 = 0.861$
$R^2 = 0.705$
$R^2 = 0.629$
$R^2 = 0.749$
$R^2 = 0.856$
Predicted proportions
Ground truth proportions
b
Priors vs. Starfysh predictions
RMSE
Signature priors
Starfysh (Signature prior)
Starfysh (Archetypal Analysis)
Starfysh (Histology Integration)
Method
c
Hyperparameter Robustness Analysis
RMSE
Prior
Starfysh
alpha
d
M.W.W. p ≤ 1e-30
M.W.W. p <= 1e-30
RMSE
JSD
BayesPrism
Cell2Location
DestVI
Tangram
BayesTME (ref-free)
CARD (ref-free)
STDeconvolve (ref-free)
Starfysh
Method
e
M.W.W. p <= 1e-30
M.W.W. p = 1.949e-09
M.W.W. p = 2.259e-11

Supplementary Figure 2. Robustness analysis of Starfysh benchmarking results on simulated data. (a)-(c) Starfysh performance and robustness analysis on 5-cell-type simulation (n = 2500 spots). (a) Reconstructed spatial distribution of inferred cell-type specific proportions (upper) and scatter plots of inferred vs. ground-truth proportions (lower). (b) Root-Mean-Square Error (RMSE) of signature priors, Starfysh with only signatures, archetypal analysis, and image integration against the ground-truth proportions. (c) Robustness Analysis of the Starfysh performance given the choice of Dirichlet strength concentration hyperparameter α. **(d)-(e)** summary of benchmarking results on simulated data (n = 2500 spots) for 5 major cell types (d) and 10 fine-grained cell types (e). Reported metrics: per-spot Root-Mean-Squared-Error (RMSE) (Left) and Jensen-Shannon Divergence (JSD) (Right) between ground-truth and predictions for each method. Box plots indicate the median (center lines), interquartile range (hinges), and 1.5x interquartile range (whiskers). A two-sided Mann-Whitney U test was performed between methods with Benjamini-Hochberg-adjusted P values.

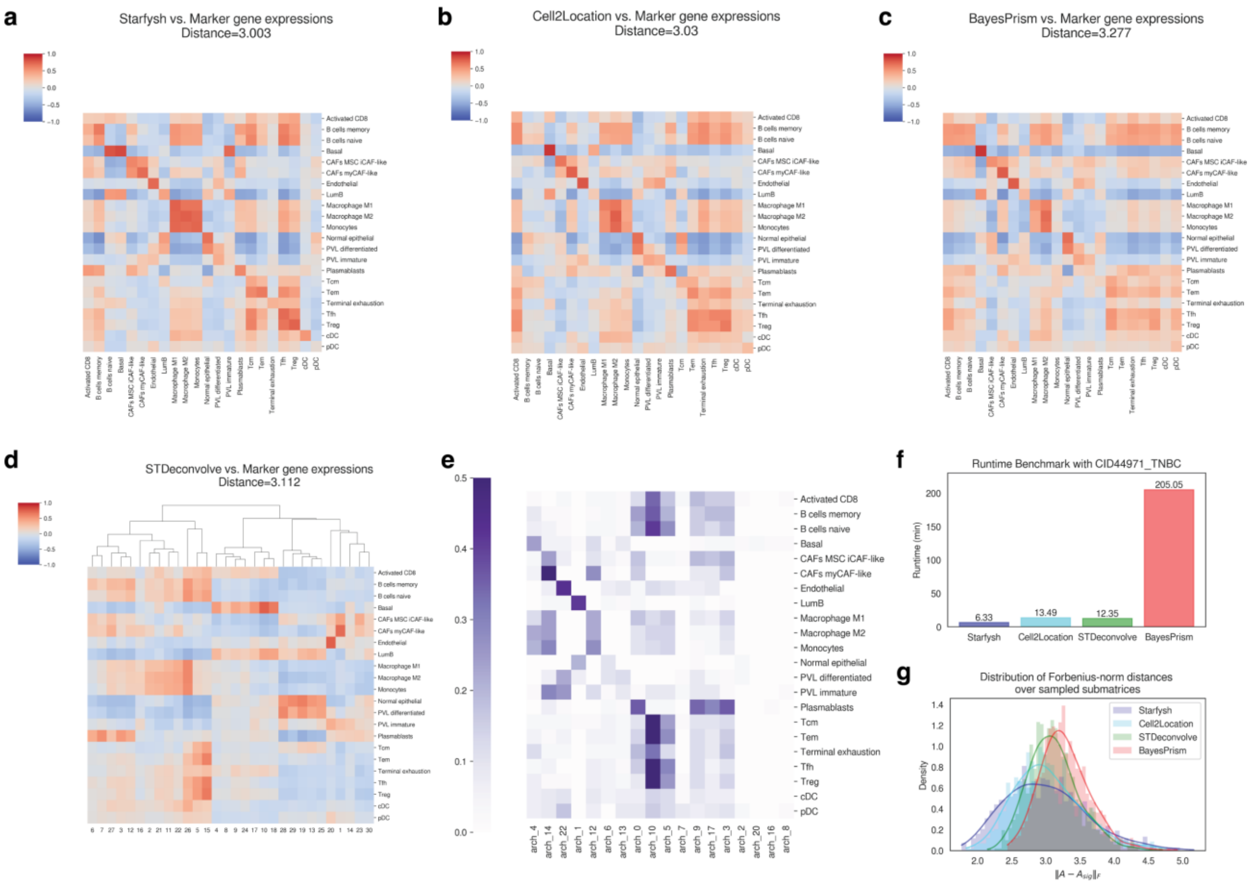

Supplementary Figure 3. Performance in disentangling refined cell states in ST data from a TNBC Patient CID44971[18] and comparison to reference-based and reference-free methods. (a)-(d) Pearson correlation computed between the normalized z-scored average expressions (y-axis) of cell-state specific genesets curated according to matched single-cell data[18] and inferred proportions (x-axis) from deconvolution (Method) using Starfysh (a), reference-based methods Cell2Location (b) and BayesPrism ©, as well as reference-free method STDeconvolve (d). The performance of each method is summarized by computing the distance between the correlation matrix and a "reference" matrix defined in Methods. Starfysh shows a significant improvement over other methods (Two-sided Mann Whitney U test on permuted cell states; p<1e-30). **(e)** Archetypal analysis without fitting Starfysh presents interpretability on major cell types but does not show interpretability and correspondence to cell states as seen with Starfysh (a) (Methods). **(f)** Runtime measured for each method applied to sample CID44971. **(g)** Distribution of Frobenius norm distance $\|A - A^{sig}\|_F$ over 1,000 sampled correlation submatrices from each method (Methods).

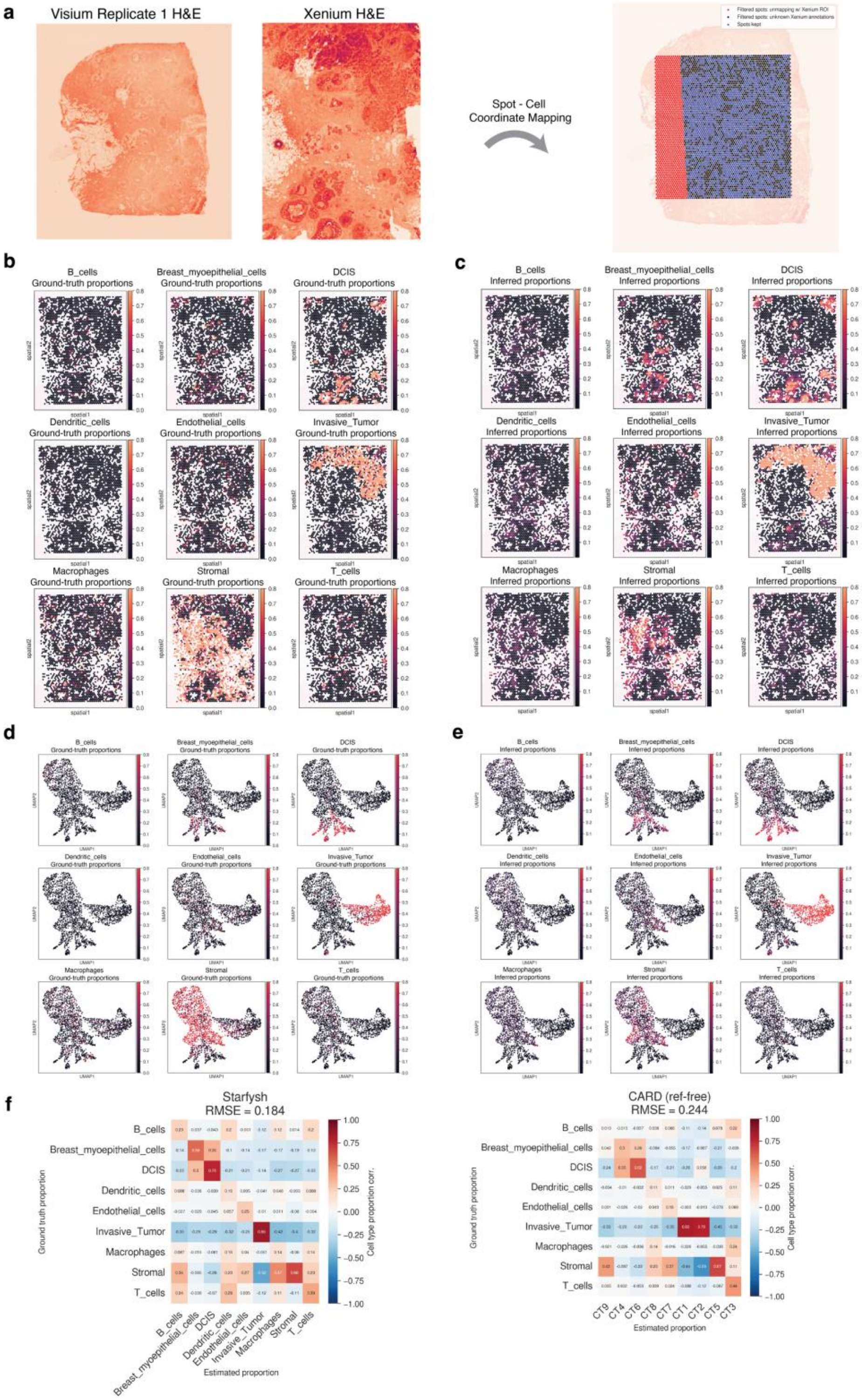

Supplementary Figure 4. Benchmarking results on ST (Visium) Breast Cancer dataset mapped with Xenium. (a) Diagram of the ground-truth cell type proportions with ST Visium-Xenium coordinate alignment. **(b)-(c)** Spatial distribution of the ground-truth (b) and Starfysh inferred (c) cell type proportions. **(d)-(e)** UMAP colored by the ground-truth (d) and Starfysh inferred (e) cell type proportions. **(f)** Benchmarking results between Starfysh (with signature priors) and representative reference-free method.

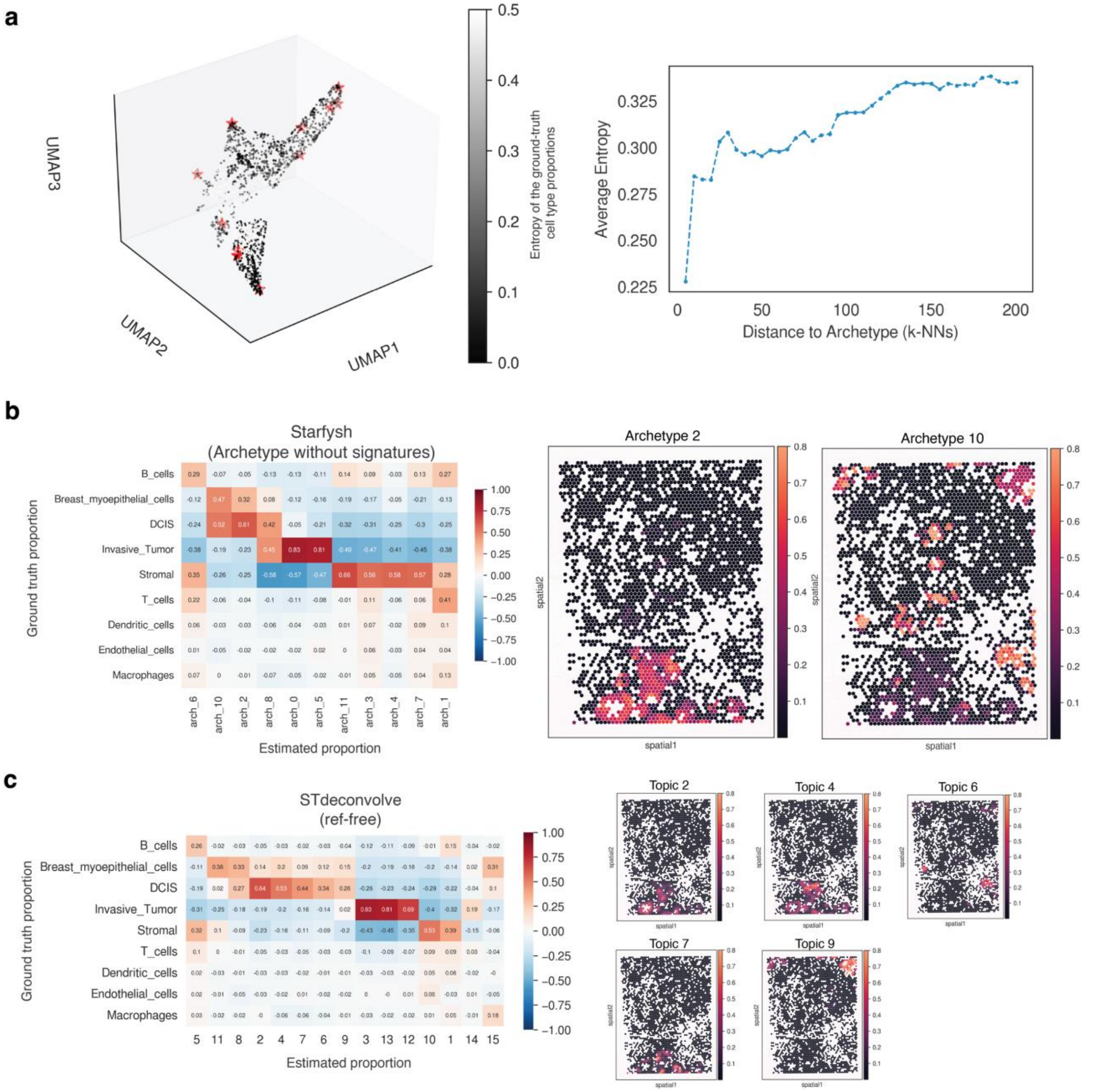

Supplementary Figure 5. Validation of Starfysh Archetypal Analysis on ST Breast Cancer dataset mapped with Xenium. (a) Archetypes are capable of identifying purest spots. Left: 3D UMAP of ST spots (applied to PC space) colored by the entropy of the distribution of ground-truth cell types in the neighborhood of spots. Archetypes are highlighted in red asterisks. Right: path length to the archetypes in the kNN graph vs. their average cell type entropy confirming lower entropy in proximity of archetypes. **(b)-(c)** Benchmarking results between Starfysh (without signature priors) and representative reference-free method. (b) Left: Heatmap of correlation between Starfysh output and the ground-truth proportions. Right: Starfysh predictions delineate two distinct DCIS subtypes. c) Left: Heatmap of correlation between STdeconvolve output and the ground-truth proportions. Right: None of a single STdeconvolve topics recovers corresponding DCIS subtypes.

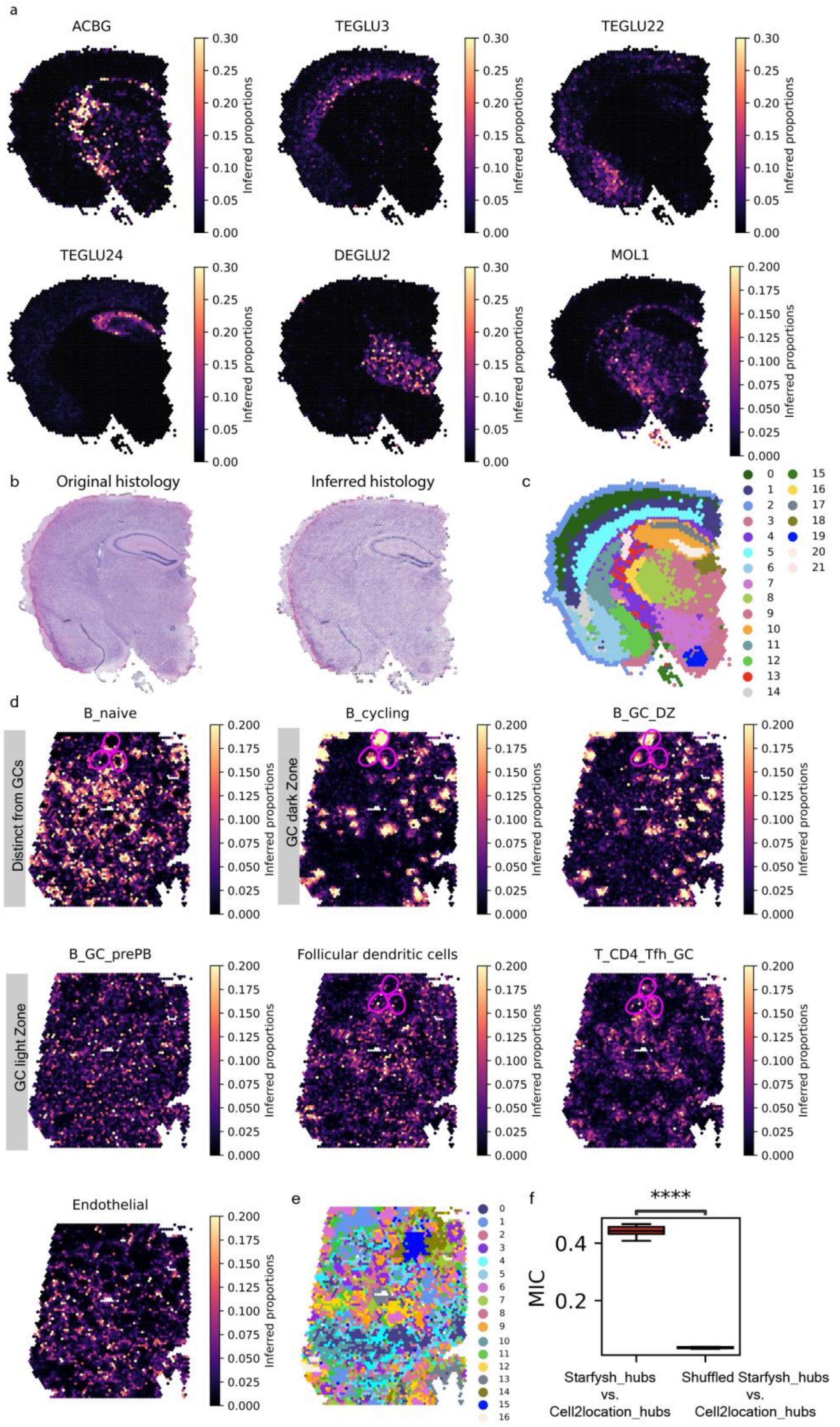

Supplementary Figure 6. Starfysh characterizes spatial mapping of cell types in mouse brain and human lymph nodes. (a) Estimated cell proportions on mouse brain. **(b)** Original histology and estimated

histology. **(c)** Spatial arrangement of brain regions identified by clustering of cell type compositions. **(d)** Estimated cell proportions in human lymph nodes. **(e)** Identified spatial hubs in human lymph nodes. **(f)** Maximum information coefficient (MIC) for alignment of Starfysh identified hubs with Cell2location identified hubs in human lymph node. Box plots indicate the median (center lines), interquantile range (hinges), and 1.5x interquartile range (whiskers). N = 10 MIC values from 1000 spots randomly sampled in tissue with human lymph nodes for both non-shuffled and shuffled hubs. One-sided independent two-sample T-test was performed. P value = 3.45e-23. ****P < 0.0001.

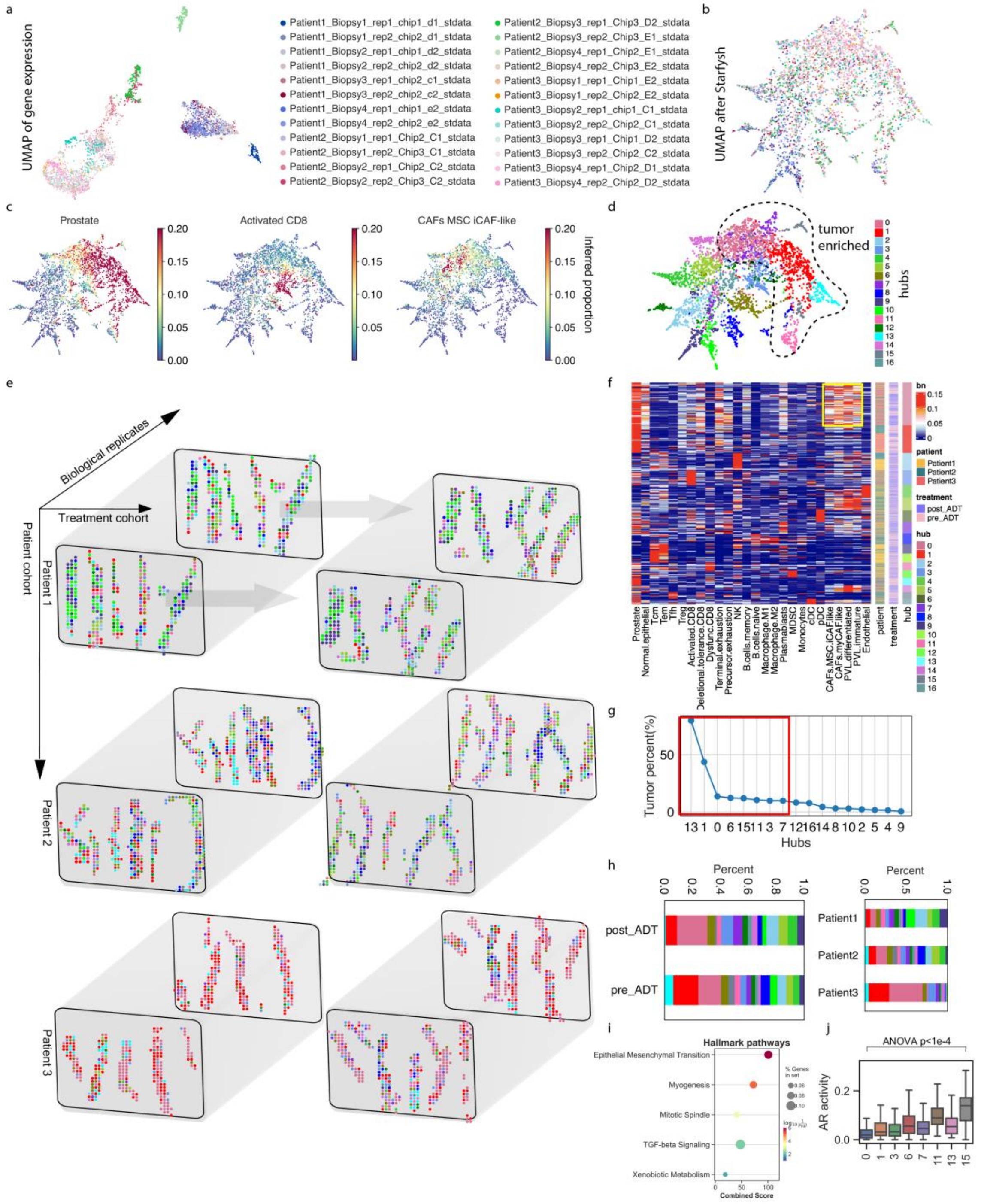

Supplementary Figure 7. Application of Starfysh to spatiotemporal data in prostate cancer. (a) UMAP visualization of gene expression across samples. **(b)** UMAP visualization of the joint latent space predicted by Starfysh, representing cell compositions. **(c)** UMAP visualization of the joint space, colored by prostate cancer types, activated CD8, and a subtype of cancer associated fibroblasts (CAFs MSC iCAF-like). **(d)** UMAP visualization of joint space of predicted cell compositions colored by identified hubs. **(e)** Spatial arrangement of hubs in tissue samples from three patients, each with 4 biopsies, 2 replicates per biopsies, and samples collected pre- and post-treatment. The colors of spots are matched with hub definitions in (d). **(f)** heatmap of inferred cell types for each spot, ranked according to the identified hubs. **(g)** Tumor percentages in the hubs. **(h)** proportions of each hub in pre- and post-treatment across patients. **(i)** Significant hallmark pathways in hub 0 (FDR<0.05, Benjamini-Hochberg) are shown with GSEA's default permutation-based test. **(j)** Androgen Receptor (AR) activity of each hub. AR activity is calculated based on the mean expression of genes in AR related pathways. Box plots indicate the median (center lines), interquartile range (hinges), and 5th to 95th percentiles (whiskers). One-way ANOVA test was performed across hubs, P value < 1e-30. N = 767, 501, 265, 208, 206, 141, 135, 112 spots in hub 0, 1, 3, 6, 7, 11, 13, and 15.

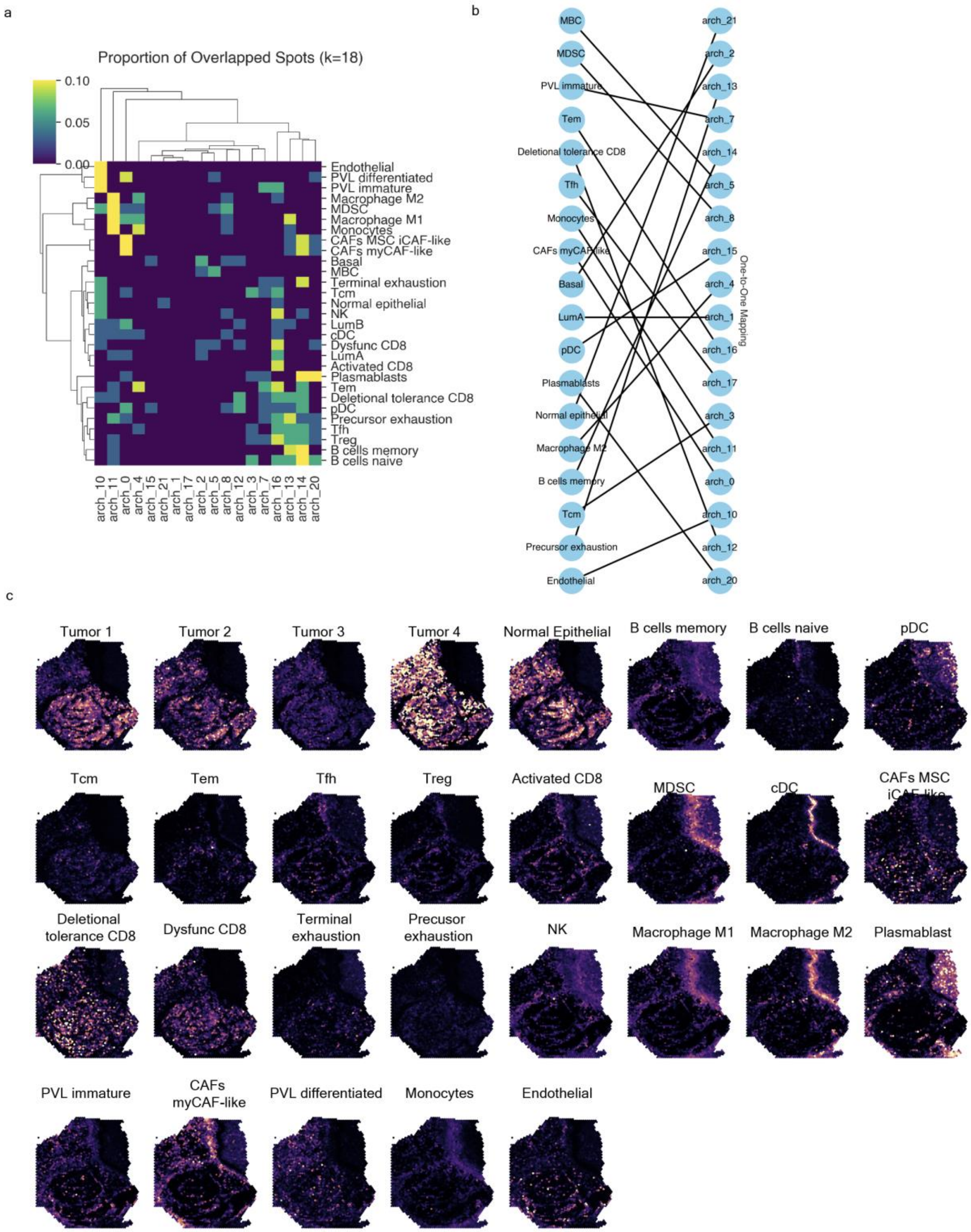

Supplementary Figure 8. Starfysh implementation on P2A_TNBC. (a) Heatmap of proportions of overlaps between spots enriched for cell types and archetypes identified in P2A_TNBC. (b) One-to-one mapping of cell types to archetypes based on provided cell type marker genes. (c) Predicted proportions of cell types by Starfysh.

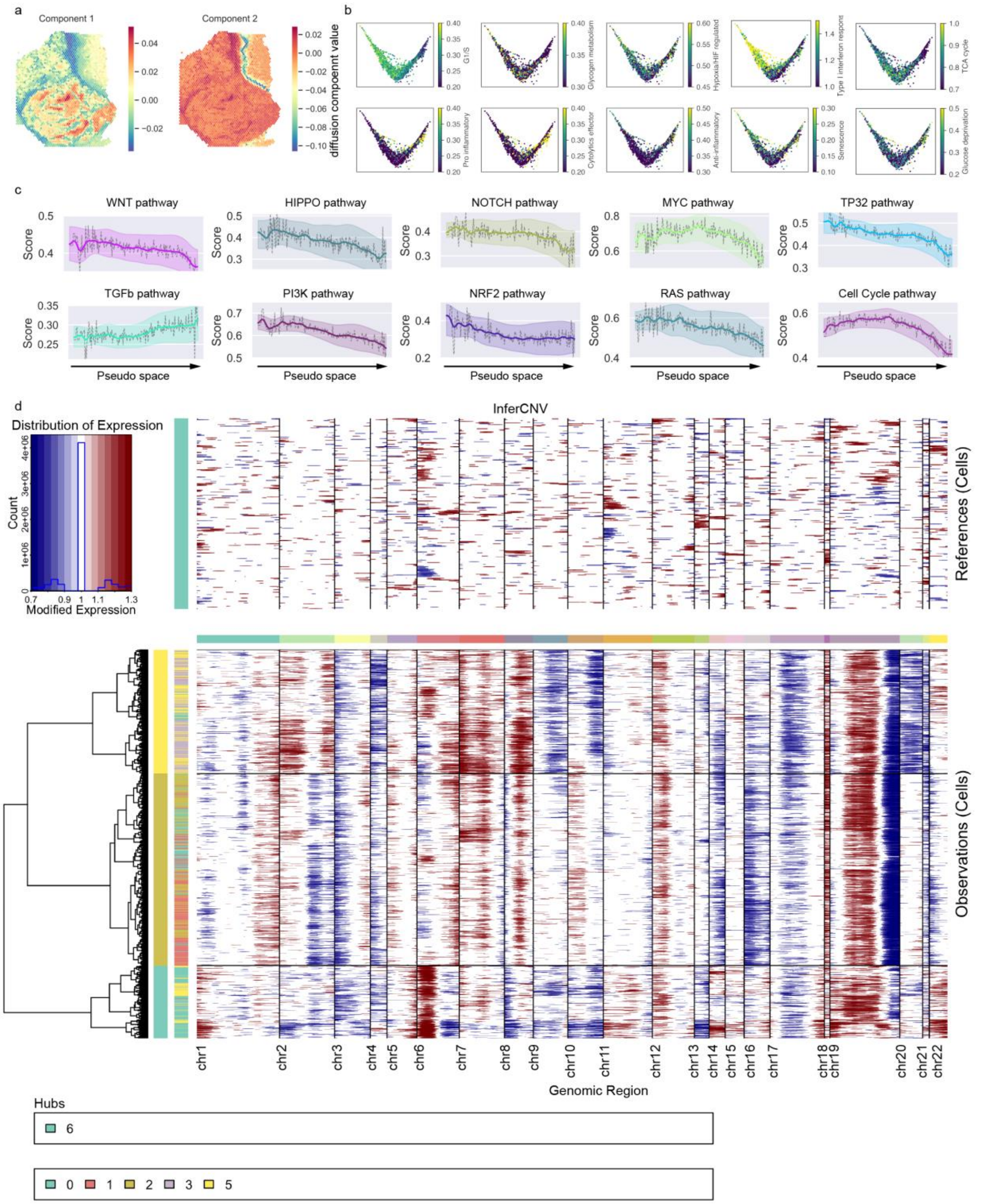

Supplementary Figure 9 Spatial heterogeneity in P2A_TNBC sample predicted by Starfysh. (a) Spatial map of diffusion components 1 and 2. **(b)** Trajectory revealed by diffusion 1 and 2 colored by metabolic signaling pathways. **(c)** Pathway expression scores (mean expression of related gene sets) along the pseudo-space axis. Data are presented as mean values +/- SD. **(d)** Heatmap of copy number variations inferred by inferCNV associated with hubs in **Fig. 2f**, with red indicating amplification and blue indicating deletion when compared to stromal hub 6 as reference. The dendrogram represents phylogeny constructed based on CNV similarity.

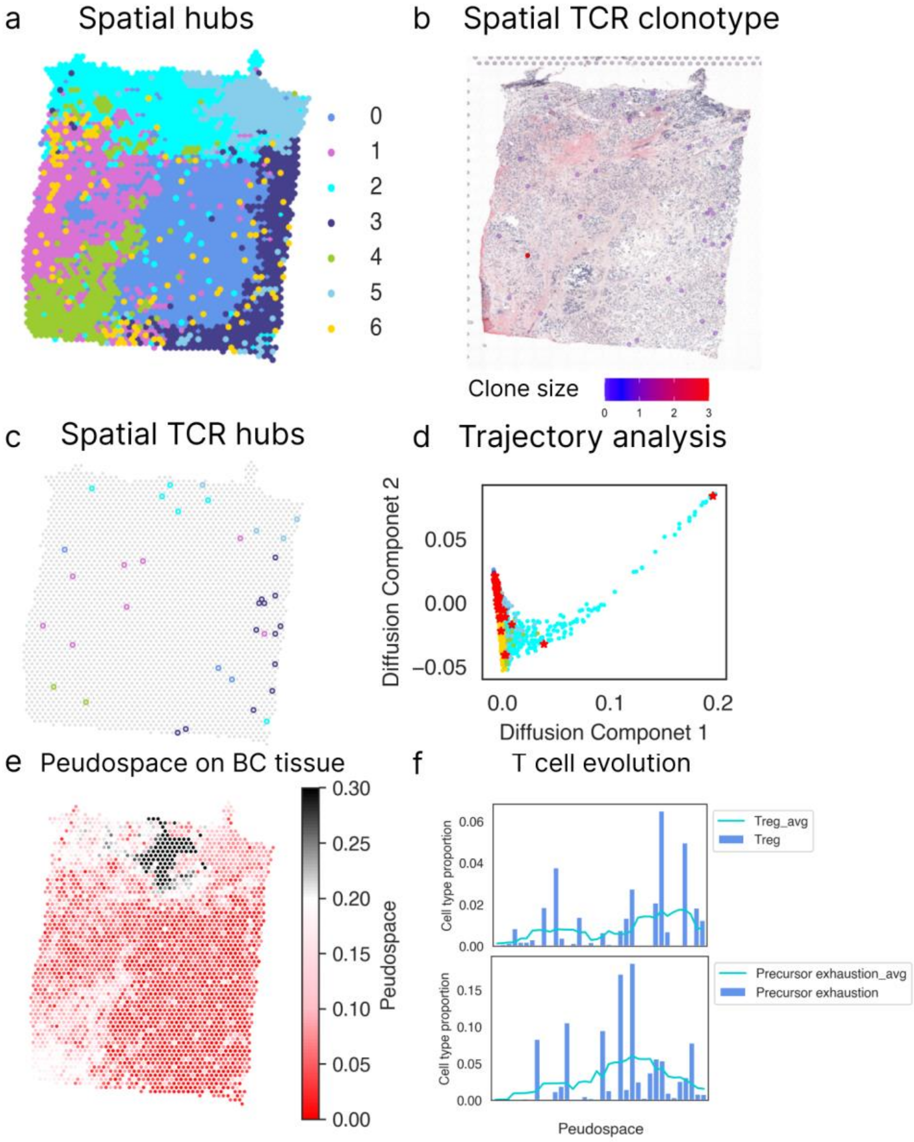

Supplementary Figure 10. Spatial T cell clonotype analysis. (a) Spatial hubs in MBC4A sample. **(b)** The largest detected clone overlaid on the histology image of MBC4A. **(c)** Hub assignments for spots within the clone shown in (b). **(d)** Trajectory analysis via diffusion map. The largest clone identified via spatial TCR sequencing is indicated in red. **(e)** Peusospace projected on the spatial map of MBC4A. **(f)** Cell type proportion in spots assigned to the same clone sorted by pseudo-space.

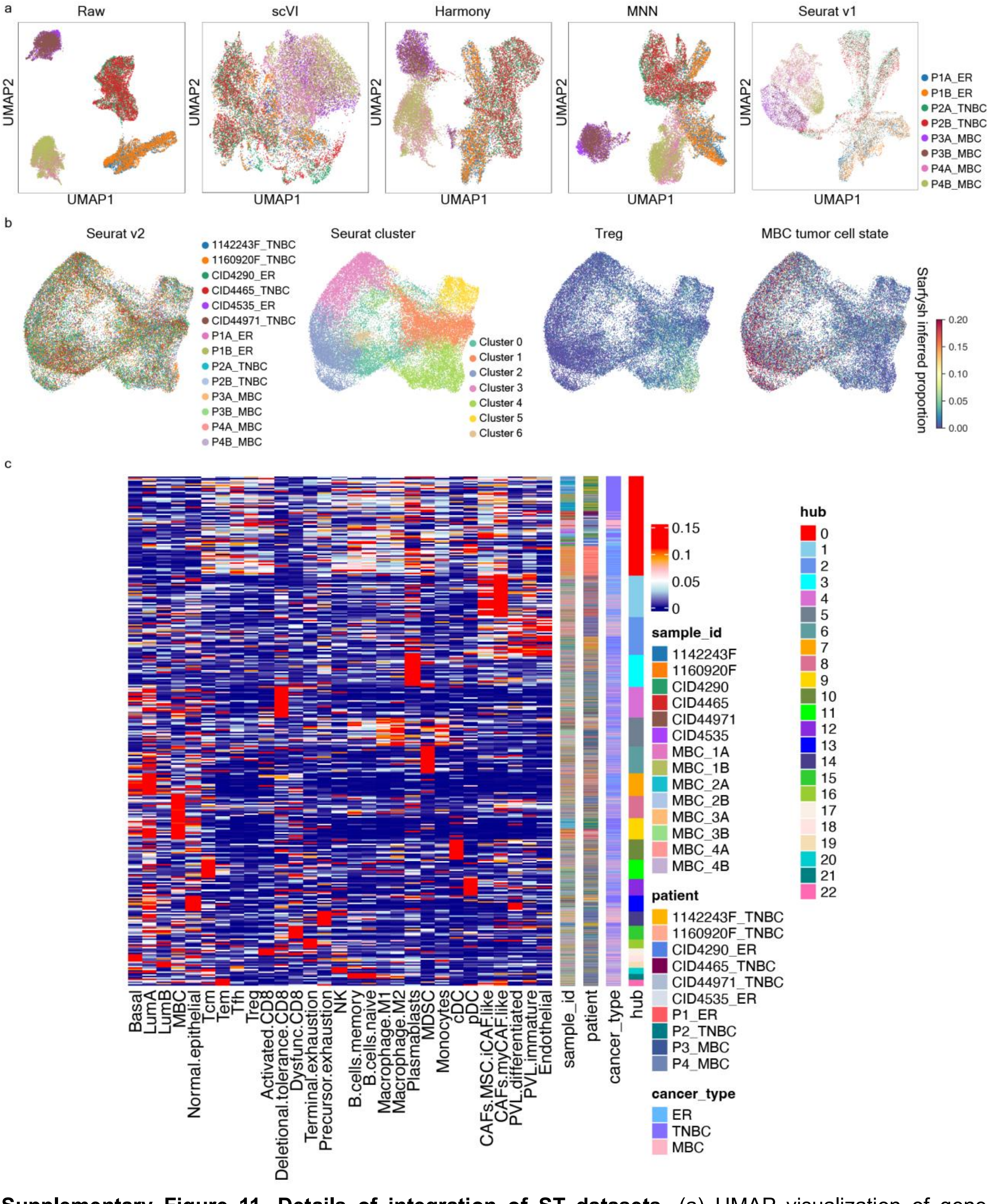

Supplementary Figure 11. Details of integration of ST datasets. (a) UMAP visualization of gene expressions of 8 samples (4 patients with 2 biological replicates each); UMAP visualization of integration with methods designed for single-cell analysis: scVI, Harmony, MNN, and Seruat v1. (b) Integrating samples with Seurat v2 (scTransform) and visualization on UMAP colored by samples, clusters, and predicted Treg, and MBC proportions by Starfysh. (c) Heatmap of clustering inferred cell types in 14 samples in the study.

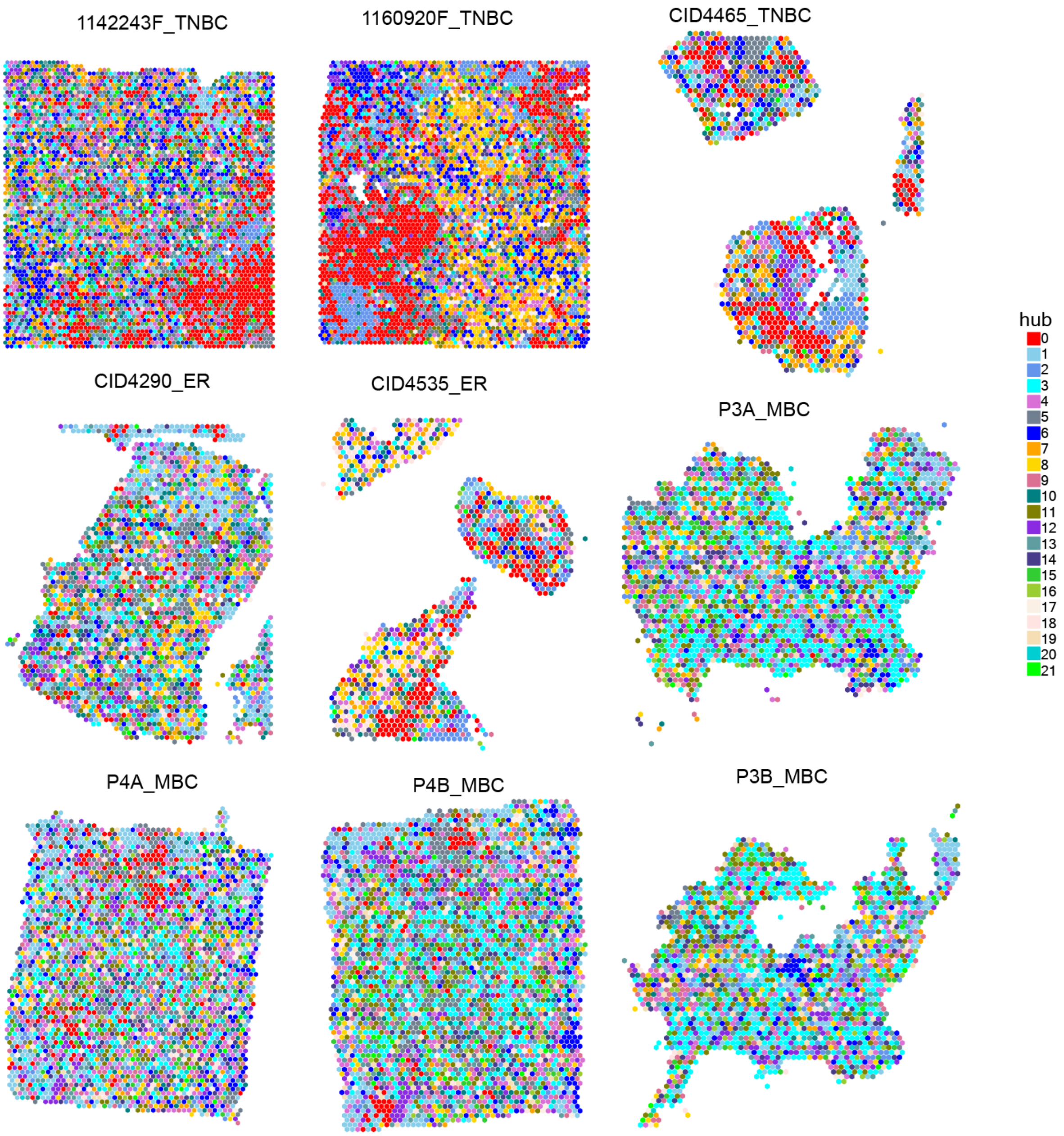

Supplementary Figure 12. Spatial arrangement of hubs for samples not shown in Fig. 3. Each spot's color corresponds to a specific hub, determined through the clustering based on the inferred proportion of Starfysh across all samples. The depicted hubs demonstrate distinct spatial patterns unique to each patient, and consistently reproducible in biological replicates within individual patients.

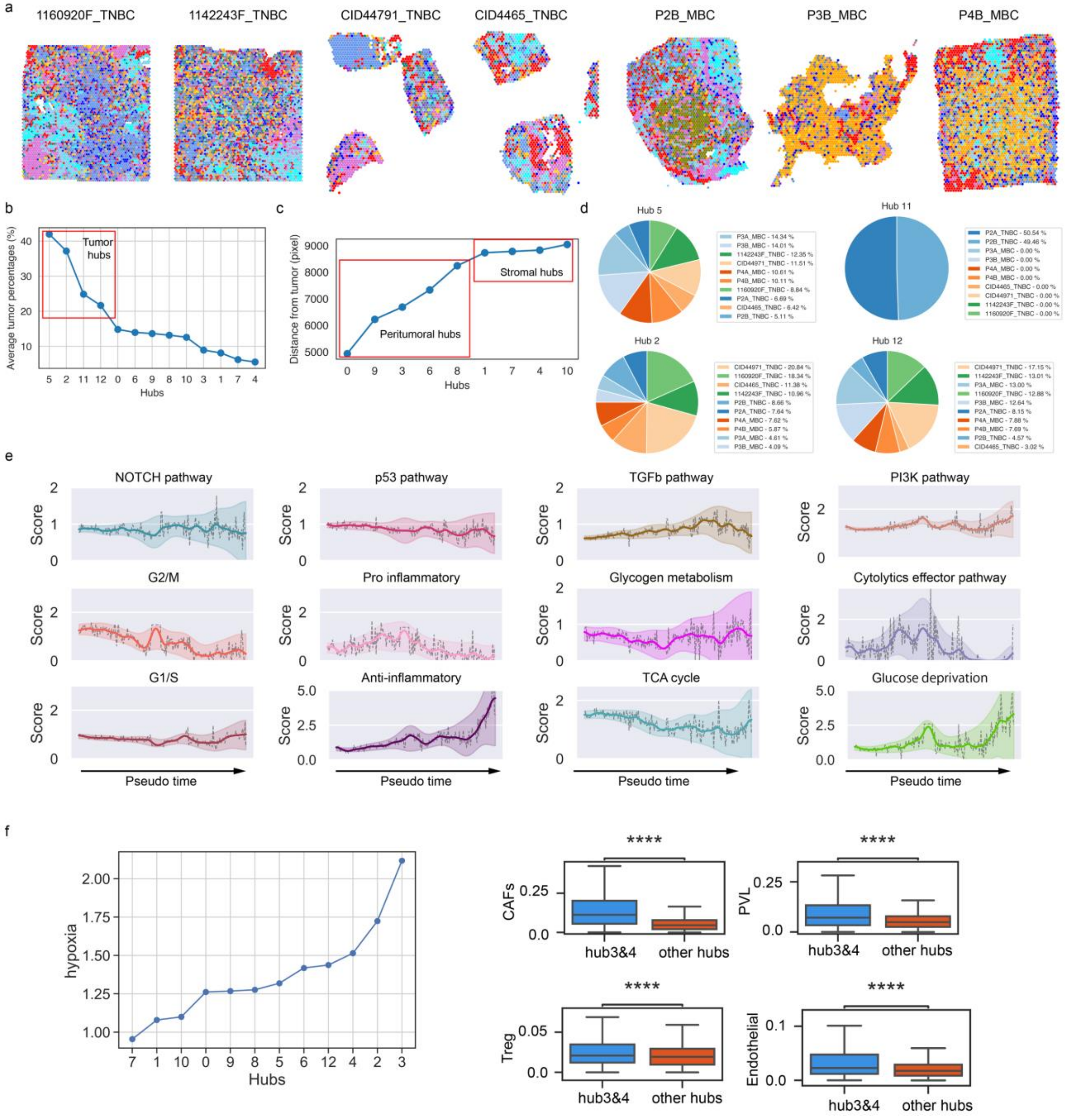

Supplementary Figure 13 Characterization of spatial heterogeneity across samples using Starfysh. (a) Spatial arrangement of hubs in TNBC and MBC samples. **(b)** Average tumor percentages across hubs. Hubs are ranked from high tumor percentages to low. Four hubs (5, 2, 11, 12) are identified as tumor hubs as shown in the red box. **(c)** Distance from tumor hubs across hubs. Hubs are ranked based on distances. Five hubs (0, 9, 3, 6, 8) are recognized as peritumoral hubs and four hubs (1, 7, 4, 10) are considered as stromal hubs. **(d)** Percentages samples in each intratumoral hub. **(e)** Metabolic signal pathways across pseudo-time identified by Starfysh. **(f)** Left panel: hypoxia across hubs. Hubs are ranked by hypoxia. The top two hubs in peritumoral and stromal regions are 3 and 4; Right panel: box plot of the CAFs, PVL, Treg, Endothelial in hub 3 and 4 compared with other hubs. Data are presented as mean values +/- SD.

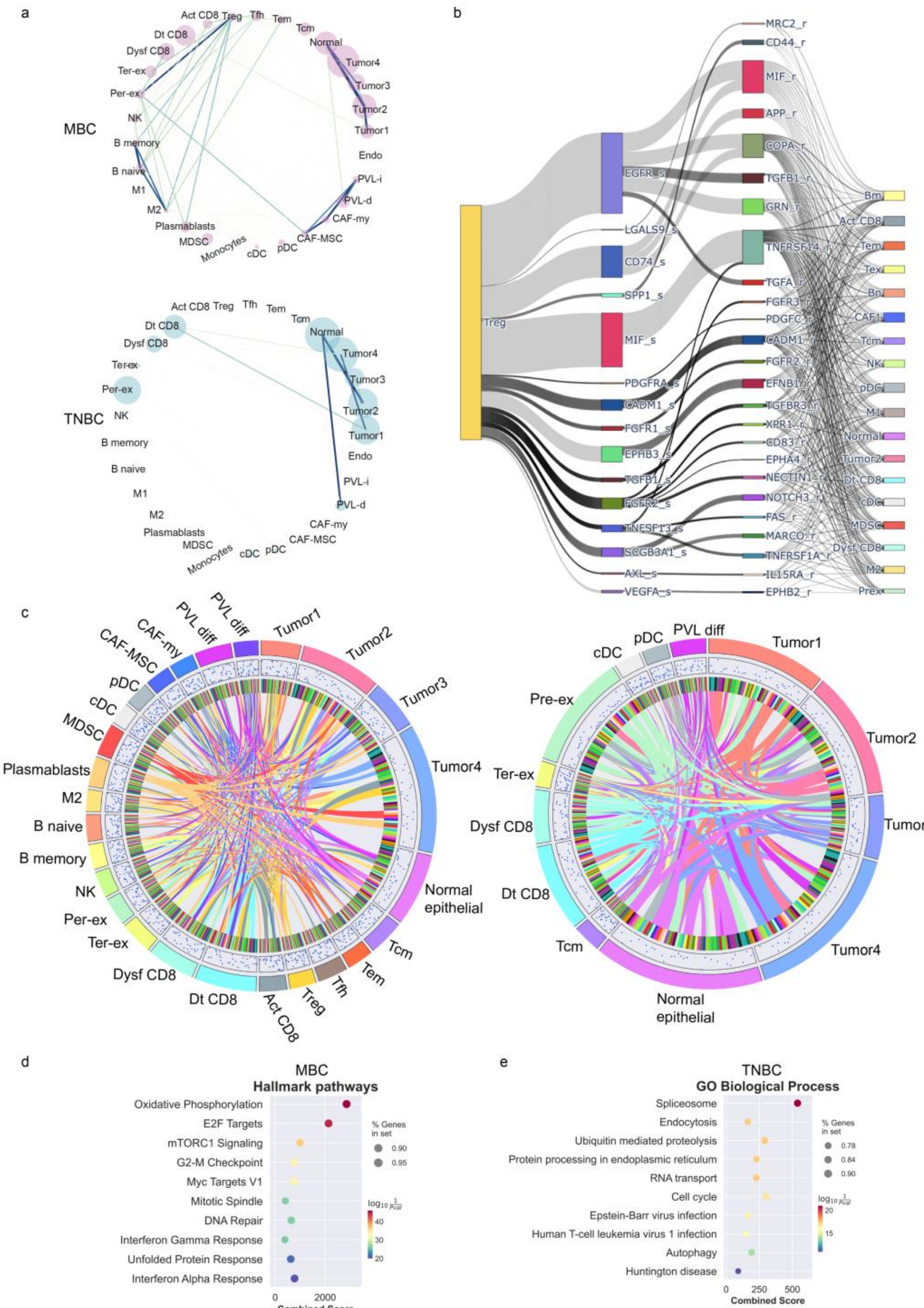

Supplementary Figure 14. Cell-cell interaction in intratumoral hubs in MBC and TNBC samples. (a) Co-localization of cell types in MBC and TNBC samples. Nodes represent the proportions of cell types, edge width is proportional to the co-localization score (SCI score, Methods). **(b)** Interaction between Treg and other cell types enriched spots through ligands and receptors inferred by cellphoneDB in the intratumoral regions in TNBC samples. **(c)** Circos plots for cell-cell interaction in MBC and TNBC samples in intratumoral regions. **(d)-(e)** Significant hallmark pathway enriched in intratumoral regions of MBC samples (d) and TNBC samples (e) at FDR<0.05 (Benjamini-Hochberg) are shown with GSEA's default permutation-based test.

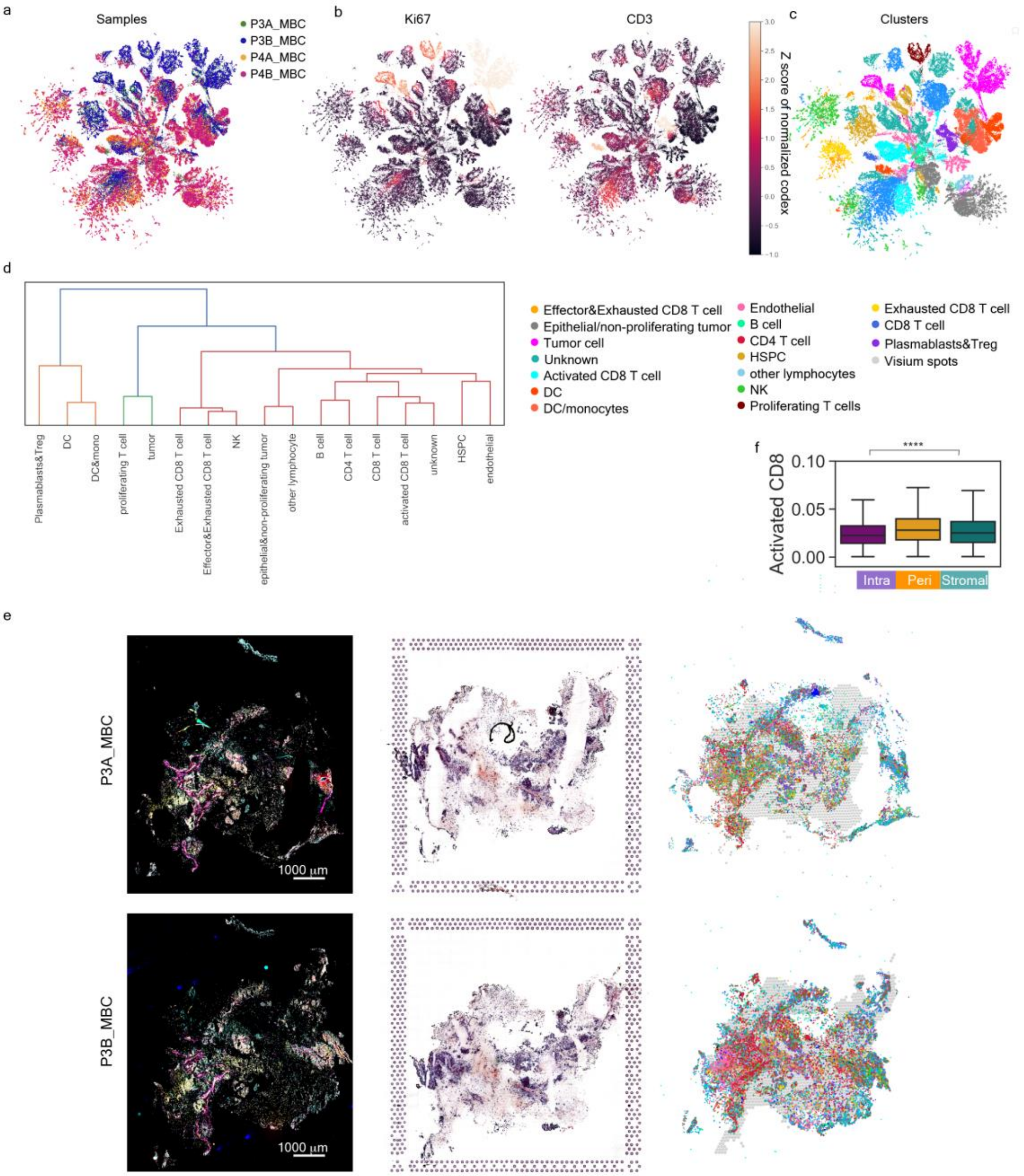

Supplementary Figure 15. Characterization of protein expression with Codex for MBC samples. **(a)** UMAP of single-cell CODEX expression data colored by samples. Each dot represents a cell. **(b)** UMAP visualization of single-cell codex data colored by Ki67 and CD3 expressions with z-score normalization. **(c)** UMAP visualization of single-cell codex data colored by clusters. **(d)** Dendrogram trees for annotated cell types. **(e)** Raw images, histology, and aligned single-cell codex with Visium spots colored by annotated cell types in P3A_MBC and P3B_MBC samples. A total of 4 MBC tissues samples were profiled, originating from 2 MBC patients with 2 biological replicates each, and 23 antibodies were used for profiling each tissue). **(f)** Barplots of expression gradients of activated CD8 T cells in intra, peri, and stromal regions. N = 1888, 7104, 1463 spots in intratumoral, peritumoral, and stromal hubs in MBC tissues. ANOVA test was performed on barplots. Box plots indicate the median (center lines), interquartile range (hinges), and 1.5x interquartile range (whiskers). One-way ANOVA test was performed. P value < 1e-30. ****P < 0.0001.
