# Supplementary material for: Starfysh integrates spatial transcriptomic and histologic data to reveal heterogeneous tumor–immune hubs
Source: Nat Biotechnol. 2024 Mar 21;43(2):223–35. doi: 10.1038/s41587-024-02173-8 (PMC11415552; doi:10.1038/s41587-024-02173-8)
Supplement: Supplementary file 2 — Reporting Summary [file 41587_2024_2173_MOESM2_ESM.pdf]

nature portfolio

Corresponding author(s): Elham Azizi, George Plitas, Alexander Y Rudensky

Last updated by author(s): Feb 1, 2024

Reporting Summary

Nature Portfolio wishes to improve the reproducibility of the work that we publish. This form provides structure for consistency and transparency in reporting. For further information on Nature Portfolio policies, see our Editorial Policies and the Editorial Policy Checklist.

Statistics

For all statistical analyses, confirm that the following items are present in the figure legend, table legend, main text, or Methods section.

n/a	Confirmed	
☐	☒	The exact sample size (*n*) for each experimental group/condition, given as a discrete number and unit of measurement
☐	☒	A statement on whether measurements were taken from distinct samples or whether the same sample was measured repeatedly
☐	☒	The statistical test(s) used AND whether they are one- or two-sided<br>*Only common tests should be described solely by name; describe more complex techniques in the Methods section.*
☐	☒	A description of all covariates tested
☐	☒	A description of any assumptions or corrections, such as tests of normality and adjustment for multiple comparisons
☐	☒	A full description of the statistical parameters including central tendency (e.g. means) or other basic estimates (e.g. regression coefficient) AND variation (e.g. standard deviation) or associated estimates of uncertainty (e.g. confidence intervals)
☐	☒	For null hypothesis testing, the test statistic (e.g. *F*, *t*, *r*) with confidence intervals, effect sizes, degrees of freedom and *P* value noted<br>*Give P values as exact values whenever suitable.*
☐	☒	For Bayesian analysis, information on the choice of priors and Markov chain Monte Carlo settings
☒	☐	For hierarchical and complex designs, identification of the appropriate level for tests and full reporting of outcomes
☐	☒	Estimates of effect sizes (e.g. Cohen's *d*, Pearson's *r*), indicating how they were calculated

Our web collection on statistics for biologists contains articles on many of the points above.

Software and code

Policy information about availability of computer code

Data collection	Software and instruments for data acquisition are outlined in the Methods section in detail. For Visium data, 10X genomics chemistries (PN 1000184), Illumina NovaSeq 6000 Reagent Kit and 3DHistech Pannoramic MIDI scanner were used for spatial transcriptomics and H&E acquisition. For Codex data, PhenoCycler (Enable lab) was used for sample preparation and staining.
Data analysis	We used Space Ranger v1.0.0 and Scanpy v1.9.2 for Visium data processing. We used Enable Medicine platform (https://www.enablemedicine.com) for Codex data processing.<br><br>Starfysh software package is publicly available at the GitHub repository https://github.com/azizilab/starfysh and has been deposited to Zenodo https://zenodo.org/records/10460548. Code for simulation, benchmarking, reproducibility and other analyses are available at the GitHub repository https://github.com/azizilab/starfysh_reproducibility. Following python packages were used for software development: numba==0.56.4, opencv-python==4.5.1, py_pcha==0.1.3, scanpy==1.9.2, scikit_dimenision==0.3, scikit_learn==1.2.1, torch==2.0.0, umap_learn==0.5.3.

For manuscripts utilizing custom algorithms or software that are central to the research but not yet described in published literature, software must be made available to editors and reviewers. We strongly encourage code deposition in a community repository (e.g. GitHub). See the Nature Portfolio guidelines for submitting code & software for further information.

Data

Policy information about availability of data

All manuscripts must include a data availability statement. This statement should provide the following information, where applicable:

- Accession codes, unique identifiers, or web links for publicly available datasets
- A description of any restrictions on data availability
- For clinical datasets or third party data, please ensure that the statement adheres to our policy

The raw data generated for this manuscript have been deposited to the National Center for Biotechnology Information's Gene Expression Omnibus (NCBI GEO) under accession number GSE218951. Public breast cancer dataset from Wu et al. are available in NCBI GEO with accession number GSE176078. Public mouse brain and lymph nodes dataset are available in ArratExpress under accession number E-MTAB-11114. Public prostate cancer dataset is available in Mendeley data (https://doi.org/10.17632/mdt8n2xgf4.1).

Human research participants

Policy information about studies involving human research participants and Sex and Gender in Research.

Reporting on sex and gender	No sex and gender information were required.
Population characteristics	All patients included in this analysis were women diagnosed and undergoing surgery for primary breast cancer (ages: P1_ER: 70yr, P2_TNBC: 84 yr, P3_MBC 71 yr, P4_MBC 52 yr). The major criteria for inclusion of specimens in this study included that the patients were treated naive and that there was sufficient material left over after standard pathologic analysis. In addition, patients were selected based on the biologic subtype of breast cancer. Gender, age or ethnicity of patients were not an exclusion factor as we did not perform analyses based on patient metadata.
Recruitment	All samples in this study were obtained from an institutional biobanking core and patients were not specifically recruited for this study. The patients had consented to an excess specimen protocol for research at the Memorial Sloan Kettering Cancer Center ensuring that the sampling did not impact patient diagnosis, treatment, or outcome. Patients were selected based on the biologic subtype of breast cancer. Gender, age, or ethnicity of patients were not an exclusion factor as we did not perform analyses based on patient metadata.
Ethics oversight	The use of patient specimens for this work was approved by the Memorial Sloan Kettering Cancer Center IRB under the following protocol numbers: 06-107, 12-206.

Note that full information on the approval of the study protocol must also be provided in the manuscript.

Field-specific reporting

Please select the one below that is the best fit for your research. If you are not sure, read the appropriate sections before making your selection.

☒ Life sciences ☐ Behavioural & social sciences ☐ Ecological, evolutionary & environmental sciences

For a reference copy of the document with all sections, see nature.com/documents/nr-reporting-summary-flat.pdf

Life sciences study design

All studies must disclose on these points even when the disclosure is negative.

Sample size	Sample size was mentioned in caption/legend.
Data exclusions	Described in the Methods section. Exclusion criteria follows the Quality Control (QC) of the data.
Replication	Biological replicates were performed by sequencing adjacent slides in tumor tissue from each patient. Data and analysis showed the reproducibility of findings. For Breast tumor Visium experiments, 2 replicates were collected per sample.
Randomization	No randomization was performed
Blinding	No blinding was performed

Reporting for specific materials, systems and methods

We require information from authors about some types of materials, experimental systems and methods used in many studies. Here, indicate whether each material, system or method listed is relevant to your study. If you are not sure if a list item applies to your research, read the appropriate section before selecting a response.

Materials & experimental systems

n/a	Involved in the study	
☐	☒	Antibodies
☒	☐	Eukaryotic cell lines
☒	☐	Palaeontology and archaeology
☒	☐	Animals and other organisms
☒	☐	Clinical data
☒	☐	Dual use research of concern

Methods

n/a	Involved in the study	
☒	☐	ChIP-seq
☒	☐	Flow cytometry
☒	☐	MRI-based neuroimaging

Antibodies

Antibodies used	Antibody panels used in CODEX data:DAPI, CD2, CD4, CD104, CD19, PD-1, CD138, CD45RO, CD21, HLA-DR, CD38, CD278, CD11c, CD31, CD3, Ki67, CD49f, CD69, CD90, CD34, Podoplanin, CD45, Pan-CK.
Validation	*Describe the validation of each primary antibody for the species and application, noting any validation statements on the manufacturer's website, relevant citations, antibody profiles in online databases, or data provided in the manuscript.*
